# Development of a next-generation chikungunya virus vaccine based on the HydroVax platform

**DOI:** 10.1371/journal.ppat.1010695

**Published:** 2022-07-05

**Authors:** Dawn K. Slifka, Hans-Peter Raué, Whitney C. Weber, Takeshi F. Andoh, Craig N. Kreklywich, Victor R. DeFilippis, Daniel N. Streblow, Mark K. Slifka, Ian J. Amanna

**Affiliations:** 1 Najít Technologies, Incorporated, Beaverton, Oregon, United States of America; 2 Division of Neuroscience, Oregon National Primate Research Center, Oregon Health and Science University, Beaverton, Oregon, United States of America; 3 Vaccine and Gene Therapy Institute, Oregon Health and Science University, Beaverton, Oregon, United States of America; 4 Division of Pathobiology and Immunology, Oregon National Primate Research Center, Oregon Health and Science University, Beaverton, Oregon, United States of America; University of Texas Medical Branch, UNITED STATES

## Abstract

Chikungunya virus (CHIKV) is an emerging/re-emerging mosquito-borne pathogen responsible for explosive epidemics of febrile illness characterized by debilitating polyarthralgia and the risk of lethal infection among the most severe cases. Despite the public health risk posed by CHIKV, no vaccine is currently available. Using a site-directed hydrogen peroxide-based inactivation approach, we developed a new CHIKV vaccine, HydroVax-CHIKV. This vaccine technology was compared to other common virus inactivation approaches including β-propiolactone (BPL), formaldehyde, heat, and ultraviolet (UV) irradiation. Heat, UV, and BPL were efficient at inactivating CHIKV-181/25 but caused substantial damage to neutralizing epitopes and failed to induce high-titer neutralizing antibodies in vaccinated mice. HydroVax-CHIKV and formaldehyde-inactivated CHIKV retained intact neutralizing epitopes similar to live virus controls but the HydroVax-CHIKV approach demonstrated a more rapid rate of virus inactivation. HydroVax-CHIKV vaccination induced high neutralizing responses to homologous and heterologous CHIKV clades as well as to other alphaviruses including Mayaro virus, O’nyong’nyong virus, and Una virus. Following heterologous infection with CHIKV-SL15649, HydroVax-CHIKV-immunized mice were protected against viremia, CHIKV-associated arthritic disease, and lethal CHIKV infection by an antibody-dependent mechanism. In contrast, animals vaccinated with Heat- or UV-inactivated virus showed no protection against viremia in addition to demonstrating significantly exacerbated CD4^+^ T cell-mediated footpad swelling after CHIKV infection. Together, these results demonstrate the risks associated with using suboptimal inactivation methods that fail to elicit protective neutralizing antibody responses and show that HydroVax-CHIKV represents a promising new vaccine candidate for prevention of CHIKV-associated disease.

## Author summary

Chikungunya virus (CHIKV) is a mosquito-borne virus that has gained significant attention due to its ability to cause large epidemics and to spread beyond endemic countries through international travelers. Despite substantial efforts over the course of many years, a licensed CHIKV vaccine remains unavailable for protecting at-risk populations. Our research group has established an advanced site-directed oxidation system, termed HydroVax, for the development of new vaccines. Here, we describe a novel CHIKV vaccine that utilizes this peroxide-based vaccine platform and demonstrates greatly improved antiviral immunity compared to other traditional virus inactivation approaches as well as complete protection against viremia, CHIKV-associated arthritic disease and lethal CHIKV infection in robust preclinical mouse models. The HydroVax-CHIKV vaccine not only induced neutralizing antibodies to geographically diverse strains of CHIKV, but also elicited neutralizing antibody responses to other clinically important alphaviruses including, Mayaro, O’nyong’nyong, and Una virus. Together, this indicates that this vaccine not only protects against CHIKV infection but may potentially provide immunity across a broader range of virulent alphaviruses as well.

## Introduction

Chikungunya virus (CHIKV) has spread globally due to the distribution and abundance of its mosquito vectors, *Aedes aegypti* and *Aedes albopictus*. In 2004, a new epidemic strain emerged in Kenya, followed by large outbreaks throughout the islands of the Indian Ocean [1]. These new epidemic strains were eventually introduced to the Western Hemisphere via infected travelers, resulting in confirmed local transmission by 2013 [1], and within a single year approximately 1.1 million cases were reported in the Americas [2], underscoring the pathogen’s explosive potential to spread within naïve human populations. Recent outbreaks of CHIKV have been associated with previously unrecognized clinical complications including severe morbidity and arthralgia that in some cases may last for years after infection and increased mortality among the elderly as well as following *in utero* transmission [3]. CHIKV infection can be lethal, with case fatality rates ranging from approximately 1 death per 1000 to 8000 reported cases [4,5], to some estimates as high as 5.7 deaths per 1000 (i.e., 1 death per 175 reported cases) when adjusted for under-reporting and patients are monitored for up to 6 months [6]. An effective vaccine that can protect the general population as well as vulnerable groups such as infants, the elderly, and the immunocompromised is clearly warranted. Although a number of CHIKV vaccines are under investigation [7], a commercial vaccine remains currently unavailable.

We [8–12] and others [13,14] have previously described a simple H_2_O_2_-based inactivation platform for the development of new and improved vaccines, including a next-generation version of this approach that incorporates a site-directed oxidation technique that provides more rapid pathogen inactivation while maintaining antigenic structures [15,16]. Here, this advanced inactivation technology was applied to the development of a CHIKV vaccine and compared to other common inactivation methodologies. Importantly, the resulting vaccine candidate, HydroVax-CHIKV, induced strong neutralizing antibodies to both homologous and heterologous/geographically-diverse CHIKV phylogenetic clades. HydroVax-CHIKV vaccination elicited neutralizing antibody responses to other alphaviruses besides CHIKV including Mayaro virus (MAYV), O’nyong’nyong virus (ONNV), and Una virus (UNAV). HydroVax-CHIKV immunized mice, as well as those that received HydroVax-CHIKV-immune sera, were protected from arthritic disease with no measurable viremia following heterologous CHIKV challenge. In contrast, vaccination of mice with CHIKV vaccines damaged by heat-inactivation or UV-inactivation resulted in no detectable neutralizing antibody responses, no protection against viremia, and caused significantly increased CD4^+^ T cell-mediated immunopathology after CHIKV infection. These experimental outcomes offer a cautionary note that development of vaccines with damaged neutralizing epitopes may result in unbalanced host immunity with the potential to exacerbate disease following subsequent exposure to the pathogen of interest. In contrast, the HydroVax vaccine platform minimizes damage to neutralizing epitopes and elicits strong, broadly reactive antiviral antibody responses that protect against CHIKV-associated arthritic disease and lethal infection. Together, these results demonstrate the feasibility of an advanced vaccine platform and provide critical proof-of-concept to support the further development of this vaccine for protection against an important emerging/reemerging mosquito-borne pathogen.

## Results

### Chikungunya virus growth and vaccine production

Based on considerations for vaccine safety and feasible commercial-scale manufacturing, the attenuated CHIKV-181/25 strain was selected for further vaccine development. This well-characterized strain was originally derived from a clinical isolate of the Asian clade (wild type strain, AF15561, GenBank EF452493), with attenuation conferred through 11 passages in African green monkey kidney cells and 18 passages in MRC-5 cells [17]. The genetic attenuation of the Biosafety Level 2 (BSL-2) CHIKV-181/25 strain provides an added layer of safety for developing an inactivated vaccine candidate while also increasing the feasibility for future large-scale cGMP production.

A cGMP-compliant Master Virus Bank (MVB) was sequenced (GenBank MW473668) and demonstrated 99.9% nucleotide identity across the sequenced region of the genome (11,992 nucleotides, 99.6% total genome coverage) in comparison to the published full-length sequence (GenBank L37661.3). The CHIKV genome structure is organized into non-structural protein (nsP1, nsP2, nsP3, nsP4) and structural protein (C, E3, E2, 6K, E1) regions, resulting in the initial translation of two polyproteins that are further processed to the individual proteins [17]. Sequencing identified a total of 4 nucleotide differences between the MVB and the CHIKV-181/25 published sequence, all within the non-structural coding region of the genome. These included 3 nonsynonymous changes (T_978_→C, A_1016_→T, G_4424_→A) resulting in amino acid differences in nsP1 (Ile_301_→Thr, Met_314_→Leu) and nsP3 (Gly_117_→Arg), as well as 1 synonymous nucleotide change (T_6043_→C). The nonsynonymous A_1016_→T and G_4424_→A differences do not appear to be reversion mutations, as they are not shared with the wild type parental strain. The nonsynonymous T_978_→C and synonymous T_6043_→C changes have been previously identified in other subclones of the CHIKV-181/25 strain and are not associated with reversion to virulence [17]. Importantly, all 4 amino acid differences were limited to the non-structural genome region, leaving the two key attenuating mutations found in the structural protein E2, which include Thr_12_→Ile and Gly_82_→Arg relative to wild-type CHIKV [17], fully maintained. To assess genetic stability, an aliquot of the MVB was expanded for 5 additional passages under GLP-compliant conditions to produce an end-of-production (EOP) virus bank. The EOP virus was sequenced (11,992 nucleotides, 99.6% total genome coverage) with no nucleotide differences observed between the MVB and EOP samples, indicating genetic stability of the virus when carried multiple passages beyond the intended production level.

**Fig 1:**
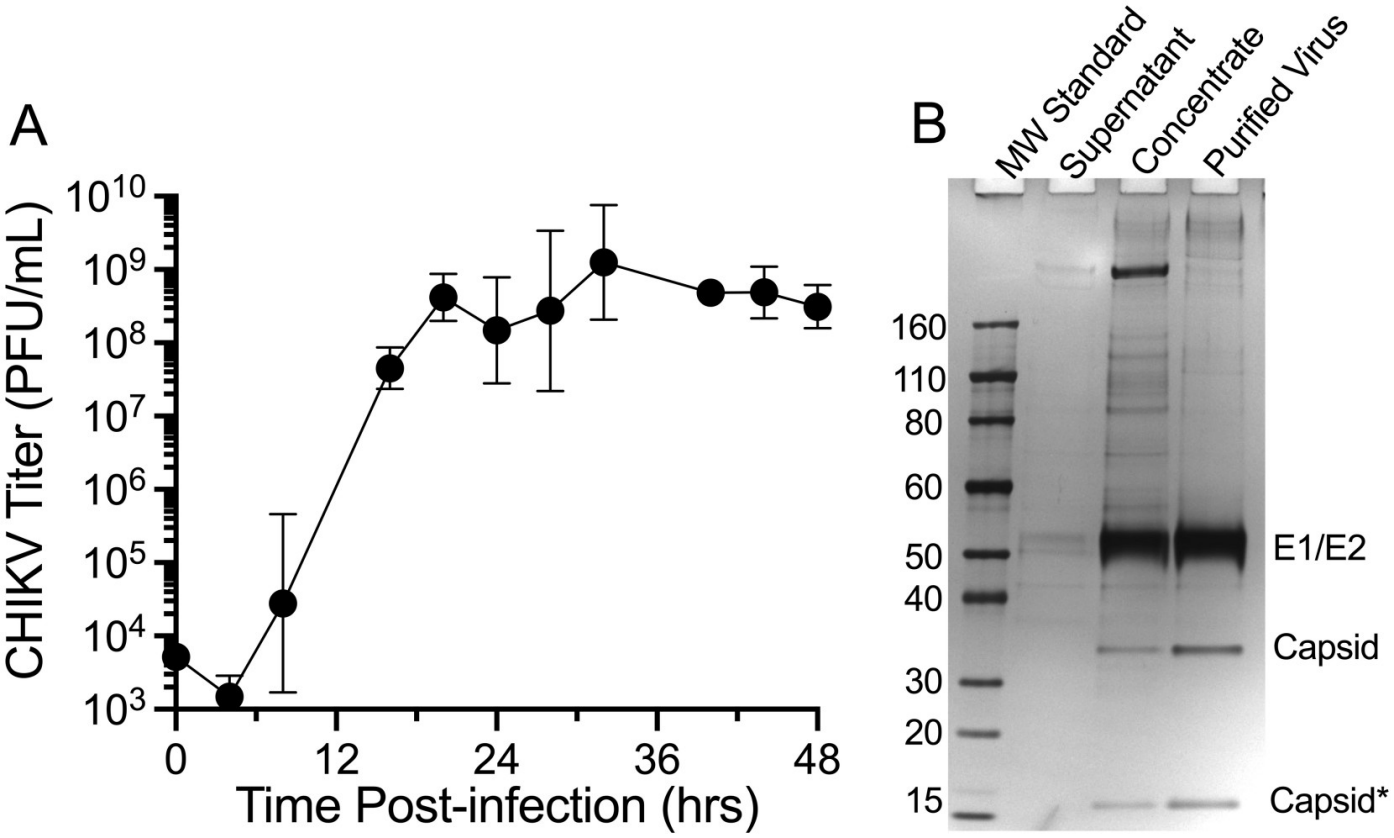
CHIKV growth kinetics and purification. (A) Growth kinetics for chikungunya virus strain 181/25 (CHIKV-181/25) were performed in triplicate in Vero cells under serum-free conditions at an MOI = 0.01. Geometric mean titers with 95% confidence intervals are shown. (B) CHIKV-181/25 supernatant was harvested at the peak of viral replication followed by concentration by tangential flow filtration (TFF) and purified virus was obtained after performing CaptoCore 700 (CC700) multimodal chromatography. Sample purity was assessed by SDS-PAGE followed by silver staining. Identity of the major protein bands in the CC700-processed sample was determined by mass spectrometry and include the co-migrating E1 and E2 envelope proteins (E1/E2), the full-length Capsid protein, as well as a band that corresponds to the Capsid C-terminal protease domain (Capsid*, [18]). The position of molecular weight markers (MW Standard) and the CHIKV structural proteins are indicated.

CHIKV-181/25 growth in serum-free Vero cells proceeded quickly, reaching peak virus titers of 10^8^−10^9^ PFU/mL by approximately 24–30 hours post-infection (Fig 1A). A single harvest of virus supernatant was performed at 30 hours post-infection and antigen purification was achieved using a previously described strategy for a HydroVax-WNV vaccine candidate [15], which includes tangential flow filtration to concentrate virus and remove lower molecular weight impurities, followed by CaptoCore 700 (CC700) multimodal chromatography to remove larger impurities. This process resulted in high-purity CHIKV antigen, with three dominant protein bands as assessed by SDS-PAGE (Fig 1B). The largest band migrated at a MW of approximately 50 kDa, with mass spectrometry analysis indicating it corresponded to the co-migrating E1 and E2 envelope proteins, each with a predicted MW of 48 kDa. The next band migrated near the 30 kDa MW standard and was identified as the C protein (predicted MW of 30 kDa) and the third protein band corresponded to the C protein C-terminal protease core domain (predicted MW of 17 kDa [18]).

### Virus inactivation and retention of neutralizing epitopes

**Fig 2:**
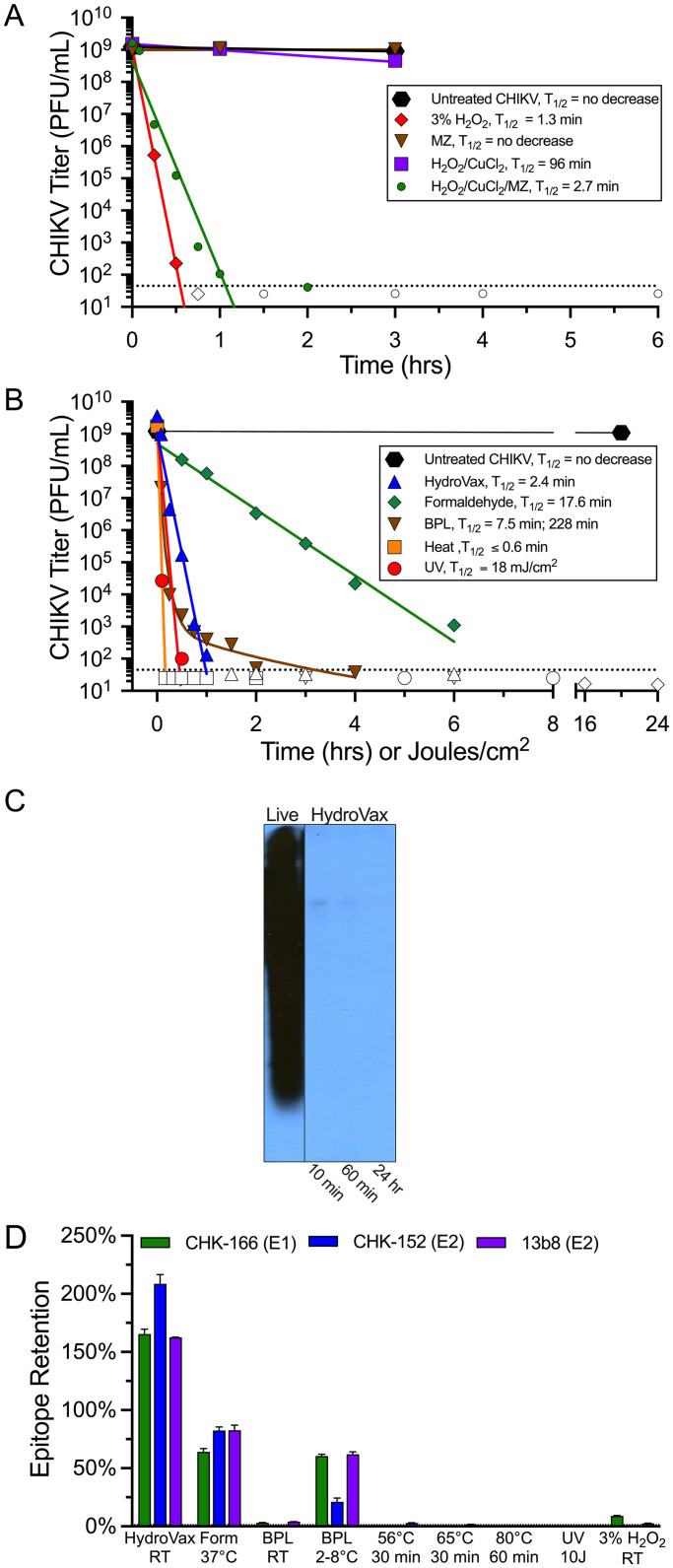
Virus inactivation kinetics and impact on CHIKV genomic RNA and neutralizing epitopes. (A) Virus inactivation kinetics based on exposure to 3% H_2_O_2_ were compared to a site-directed dual oxidation approach (H_2_O_2_/CuCl_2_/MZ) and selected subcomponents of the site-directed dual oxidation system at matched concentrations (H_2_O_2_/CuCl_2_ or MZ). (B) Kinetics of virus inactivation based on an optimized HydroVax vaccine approach were compared to other common virus inactivation methods including 0.01% formaldehyde, 0.10% BPL, heat inactivation at 80°C, and UV irradiation. Inactivation studies were performed at room temperature except for formaldehyde, which was performed at 37°C and heat inactivation, which was performed at 80°C. The geometric mean titer of virus from duplicate or triplicate experiments are presented. The dotted line indicates the limit of detection and open symbols signify time points when no detectable live virus was observed. Inactivation kinetics were modeled using first-order or second-order (BPL only) decay rate equations, resulting in the indicated T_1/2_ values. (C) Northern blot analysis was performed using a CHIKV genome-specific probe. Purified CHIKV-181/25 was left untreated (Live) or treated with the HydroVax approach for the indicated periods of time, followed by extraction of RNA from matched amounts of CHIKV-181/25. (D) Purified CHIKV-181/25 was inactivated with the optimized HydroVax approach (20 hours at room temperature), formaldehyde (0.01% for 20 days at 37°C), BPL (0.10% for 20 hours at room temperature or 2–8°C), heat inactivation (30 min at 56°C, 30 min at 65°C or 60 min at 80°C), ultraviolet irradiation (UV, 10J) or high concentration hydrogen peroxide (3% H_2_O_2_, 20 hours at room temperature). Maintenance of neutralizing antibody binding sites were compared to freshly thawed live purified CHIKV by ELISA using CHIKV E1-specific MAb CHK-166, CHIKV E2-specific MAb CHK-152, or the CHIKV E2-specific MAb 13b8. Abbreviations: MZ; methisazone, Form; formaldehyde, BPL; β-propiolactone, UV; ultraviolet, mJ; millijoule, J; joule, RT; room temperature.

Following the establishment of a suitable manufacturing process for preparing high-purity CHIKV vaccine antigen, we next explored different virus inactivation approaches for candidate vaccine development (Fig 2). We developed an advanced oxidation approach [15,16] based on Fenton-type chemical reactions where ions of the redox-active transition metal, copper (Cu), act as a catalyst that interacts with H_2_O_2_ to produce hydroxyl radicals when bound to macromolecules [19]. Importantly, to focus the specificity of the oxidative reaction on nucleic acid targets, we incorporated the antiviral drug, methisazone (MZ), into the process [15]. MZ binds Cu [20] and these complexes are known to bind nucleic acids [21]. Together, this strategy improves the kinetics of H_2_O_2_-based inactivation by approximately 35-fold compared to H_2_O_2_ + Cu-based inactivation in the absence of MZ while simultaneously preserving neutralizing epitopes on protein antigens and preventing the damage observed during inactivation with 3% H_2_O_2_ alone [15] (S1 Fig). Based on pilot optimization studies, we developed a lead inactivation formulation containing 0.0003% H_2_O_2_, 2 μM CuCl_2_, and 20 μM MZ. We assessed how the different components of this inactivation approach influenced the rates of virus inactivation in comparison with our original inactivation approach based on using 3% H_2_O_2_ (Fig 2A). Untreated live CHIKV showed little to no loss in virus titer over the course of the experiment whereas addition of 3% H_2_O_2_ led to rapid virus inactivation (T_1/2_ = 1.3 minutes). Incubation of CHIKV with MZ alone showed no change in virus titer, indicating that MZ does not have intrinsic antiviral activity in vitro. The combination of 0.0003% H_2_O_2_ with 2 μM CuCl_2_ demonstrated slow virus inactivation kinetics with a half-life estimated at T_1/2_ = 96 minutes. However, addition of MZ to the H_2_O_2_/CuCl_2_ cocktail resulted in rapid virus inactivation (T_1/2_ = 2.7 minutes) that was nearly equivalent to that observed with a 10,000-fold higher concentration of 3% H_2_O_2_. Interestingly, although exposure to 3% H_2_O_2_ resulted in the complete ablation of neutralizing epitopes, the H_2_O_2_/CuCl_2_/MZ combination rapidly inactivated CHIKV while maintaining the structural integrity of neutralizing epitopes (S1 Fig). Together, this shows that addition of MZ not only increases the speed of virus inactivation, but also demonstrates greatly improved site-specificity of the oxidation reaction since this form of inactivation was not accompanied by the oxidative damage to protein epitopes observed following incubation with 3% H_2_O_2_ alone. To further improve the performance of the H_2_O_2_/CuCl_2_/MZ combination, we added a stabilizing concentration of polyatomic oxyanions (150 mM NaPO_4_) that reduce damage to neutralizing epitopes during inactivation [22] and 0.06% formaldehyde since prior studies have indicated that brief exposure to formaldehyde can enhance protein stability and subsequent vaccine-induced immunity [23]. Addition of formaldehyde to the final HydroVax inactivation formula did not appreciably change inactivation kinetics (T_1/2_ = 2.4 minutes, Fig 2B), indicating that the rapid rates of virus inactivation (T_1/2_ = 2.7 minutes, Fig 2A) are driven mainly by site-directed oxidation.

In addition to the optimized HydroVax system, we also compared inactivation kinetics of other common inactivation approaches used in laboratory settings or for vaccine development including treatment with formaldehyde, BPL, heat, or UV irradiation (Fig 2B). For chemical inactivation approaches, we employed published methods used for virus inactivation by formaldehyde (0.01% formaldehyde, 20 days at 37°C [24]) and BPL (0.10% BPL, overnight at room temperature [25]). Formaldehyde-based inactivation kinetics were the slowest (T_1/2_ = 17.6 minutes) and this explains why extended incubation times involving several days or weeks are often required to mitigate concerns over tailing of the inactivation kinetics that could potentially result in residual live virus [26]. BPL is known to quickly hydrolyze in aqueous solutions and lose inactivating potential [25] and we observed a similarly rapid loss in inactivation potential over a short period of time with CHIKV. BPL-based CHIKV inactivation kinetics were biphasic and were modeled with both fast (T_1/2_ = 7.5 minutes) and slow (T_1/2_ = 228 minutes) half-lives, with an inflection point indicative of a substantial decrease in the inactivation rate observed approximately 1 hour after initiation of the reaction. For heat inactivation, preliminary studies using temperatures of either 56°C or 65°C indicated that a small fraction of CHIKV was heat resistant with low levels of infectious virus still detected at 25–30 minutes after initiating heat-based inactivation (S2 Fig). These conditions would be unsuitable for inactivated vaccine development purposes due to the potential risk of residual live virus. By contrast, heat inactivation at 80°C demonstrated a rapid and linear rate of inactivation with T_1/2_ ≤0.6 minutes and UV irradiation was likewise very efficient with a T_1/2_ = 18 mJ/cm^2^.

To determine if oxidative damage to CHIKV RNA was a likely mechanism of action (MOA) underlying HydroVax-based virus inactivation, we extracted RNA from purified live CHIKV-181/25 (1.4x10^9^ PFU equivalents) and compared it to CHIKV RNA extracted in parallel from an equal amount of the same lot of purified CHIKV-181/25 that had been inactivated for 10 minutes, 60 minutes, or 24 hours using the HydroVax platform before samples were analyzed by Northern blot using a CHIKV-specific probe [27] (Fig 2C). Although CHIKV RNA from purified live virus provided an intense signal, within as little as 10 minutes after HydroVax-based inactivation, CHIKV genomic RNA was degraded to barely detectable levels by Northern blot analysis. This rapid destruction of intact CHIKV genomic RNA is consistent with the rapid loss of viable infectious virus observed in Fig 2B.

In terms of vaccine development, the rates of virus inactivation are secondary only to the maintenance of structural integrity and the retention of neutralizing epitopes since this is critical for providing the most immunogenic vaccine formulation *in vivo*. Retention of neutralizing epitopes was assessed in comparison with untreated live virus incubated under similar conditions in parallel to the various inactivation conditions described here, as measured by the binding of three high affinity neutralizing MAbs mapped to both the E1 and E2 structural proteins (Fig 2D). These include CHK-166 (E1-specific, [28]), CHK-152 (E2-specific, [28]), and 13b8 (E2-specific). The HydroVax approach provided the highest retention of neutralizing epitopes after inactivation whereas formaldehyde-based inactivation resulted in epitope retention that was approximately half of that observed with the HydroVax technique. BPL-based inactivation, at both room temperature and 2–8°C, resulted in substantial damage to neutralizing epitopes. Although inactivation with BPL at 2–8°C was less damaging than inactivation performed at room temperature, prior studies with influenza virus also observed substantial antigenic damage as well as cases of breakthrough of live virus when inactivation was performed at 2–8°C [29]. Each of the heat inactivation conditions (56°C, 65°C, and 80°C) as well as UV-based inactivation resulted in complete ablation of neutralizing epitopes. High concentration (3%) H_2_O_2_ treatment was likewise highly damaging. To determine if the differences in epitope retention observed after virus inactivation might impact vaccine potency and induction of neutralizing antibody titers, the immunogenicity of representative vaccine formulations were tested *in vivo*.

### Vaccine-induced CHIKV-specific antibody responses

**Fig 3:**
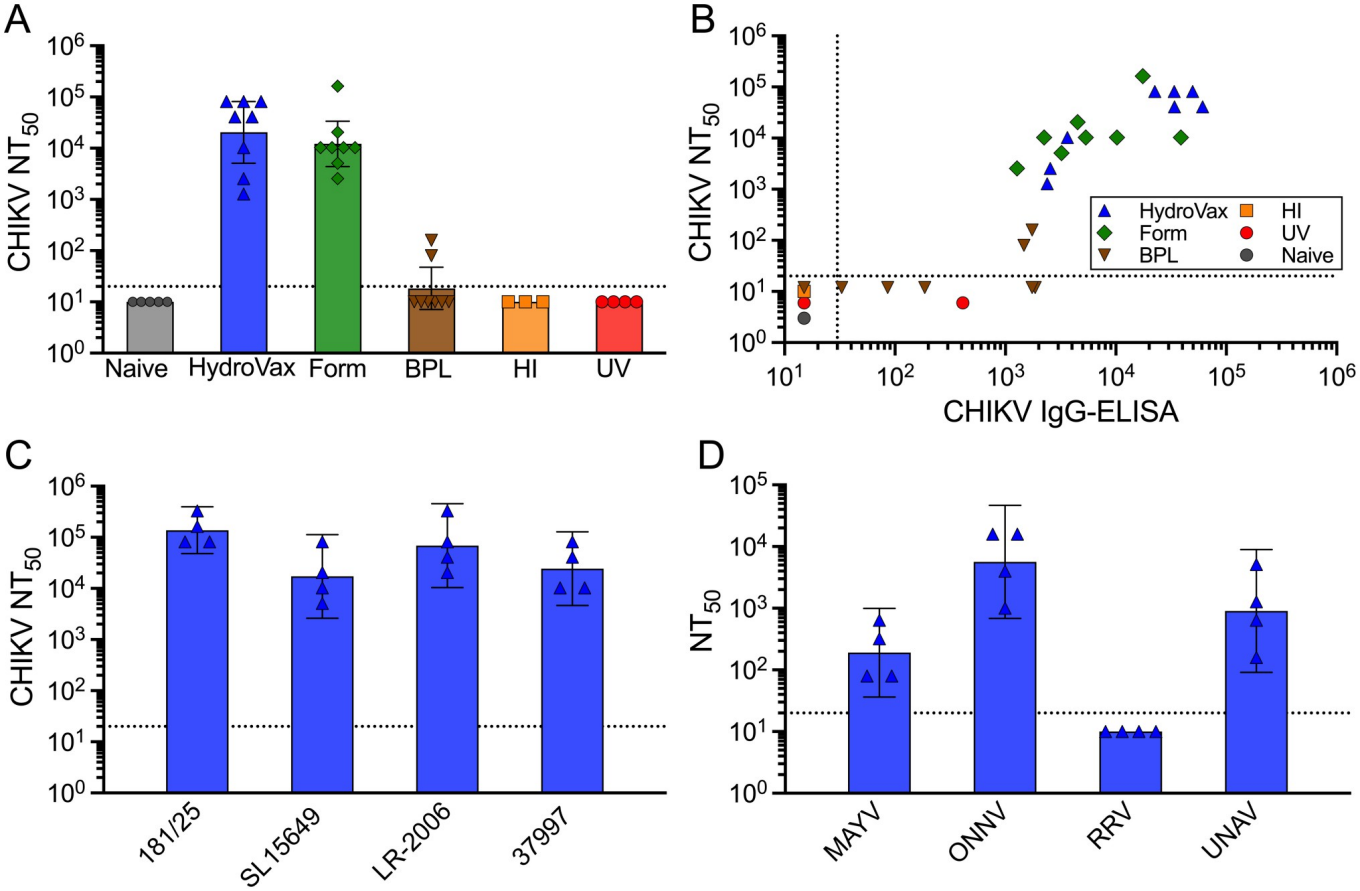
HydroVax-CHIKV induces antibody responses against heterologous CHIKV strains in addition to other arthritogenic alphaviruses including MAYV, ONNV, and UNAV. (A) Purified CHIKV-181/25 was inactivated with the optimized HydroVax approach (20 hours at room temperature), formaldehyde (Form., 0.01% for 20 days at 37°C), β-propiolactone (BPL, 0.10% for 20 hours at room temperature), heat inactivation (HI, 1 hour at 80°C) or ultraviolet irradiation (UV, 10J). C57BL/6 mice (*n* = 5–8 per group) were immunized on days 0 and 28 with 0.5 μg of the optimized HydroVax-CHIKV, 0.5 μg of Formaldehyde-inactivated virus, a 2 μg dose of BPL-inactivated virus or a 5 μg dose for heat-inactivated or UV-irradiated virus. Serum samples were collected on day 56 and assessed for vaccine-homologous CHIKV-181/25 neutralizing activity. (B) Serum samples were assessed for neutralizing activity and virus-binding antibody titers to CHIKV-181/25 as determined by ELISA. Neutralizing titers below the limit of detection (NT_50_<20) were given arbitrary values to allow visualization of individual symbols within the graph. (C) Mice immunized with HydroVax-CHIKV on days 0 and 28 (ranging from 0.8–2 μg per dose) were tested for homologous and heterologous CHIKV-specific neutralizing activity at 56 days following the primary vaccination (28 days following booster vaccination). (D) The serum samples examined in panel C were tested for neutralizing activity against Mayaro virus (MAYV), O’nyong’nyong virus (ONNV), Ross River virus (RRV), and Una virus (UNAV). Each individual data point is shown along with the geometric mean represented by a bar and 95% confidence intervals. Dotted lines represent the limit of detection.

The HydroVax-CHIKV vaccine candidate was compared to two commonly used commercial-scale inactivating agents, formaldehyde and BPL, as well as two common laboratory approaches to virus inactivation including heat inactivation and UV irradiation. HydroVax-CHIKV and formaldehyde-treated vaccine formulations were administered to C57BL/6 mice at a 0.5 μg dose in a two-dose series administered on days 0 and 28. At day 56 (i.e., 28 days after booster vaccination), the geometric mean titer (GMT) of neutralizing antibodies reached 20,500 (95% CI: 5,100–81,800) among animals that received HydroVax-CHIKV compared to a GMT of 12,200 (95% CI: 4,400–33,600) among animals that received formaldehyde-inactivated virus. Mice that were vaccinated at day 0 and 28 with a 0.5 μg dose of BPL-inactivated CHIKV failed to seroconvert and therefore the experiment was repeated with a 2 μg dose. Under these conditions, only 2/8 BPL-CHIKV vaccinated animals seroconverted, and both animals had neutralizing titers that were ≤ 160 (Fig 3A). In consideration of the complete loss of neutralizing epitopes on virus damaged by heat- or UV-based inactivation (Fig 2C), a 5 μg dose of antigen was utilized for these two vaccines to improve the chance of detecting neutralizing antibody responses among the vaccinated animals. However, neither heat-inactivated nor UV-inactivated CHIKV vaccines elicited measurable neutralizing antibody responses (NT_50_ < 20, Fig 3A).

High quality antibody responses often show a good correlation between neutralizing antibody titers and total virus-binding antibody titers as measured by ELISA. To determine the quality of the antibody response elicited by the various CHIKV vaccine candidates described here, functional neutralizing antibody titers were plotted versus total virus-binding IgG ELISA titers (Fig 3B). Naïve control sera from unvaccinated mice scored below the limit of detection for both assays. Serum from heat-, UV-, or BPL-CHIKV vaccinated animals generally scored below detection by either approach, although a few animals mounted weak ELISA responses despite having little or no measurable neutralizing antibodies. This result might be expected based on the substantial damage caused during the inactivation process (Fig 2C). By comparison, both HydroVax-CHIKV and formaldehyde-inactivated CHIKV induced balanced antibody responses that showed a linear correlation between CHIKV-neutralizing titers and virus-binding serum antibody levels by ELISA.

All CHIKV strains belong to a single serotype but there are four genetically and geographically distinct clades of CHIKV, including the Asian, East/Central/South African (ECSA), West African, and Indian Ocean Lineage (IOL) clades [7]. CHIKV-181/25 is from the Asian clade [17]. CHIKV-SL15649 was selected as a representative of the ECSA clade while CHIKV-37997 was chosen from the West African clade [30]. The La Reunion strain (CHIKV-LR-2006) is considered an epidemic Indian Ocean Lineage (IOL) clade member [1]. As shown in Fig 3C, the HydroVax-CHIKV vaccine elicited neutralizing responses against each CHIKV clade. To determine if HydroVax-CHIKV elicits antibody responses to other alphaviruses, we measured neutralizing antibody titers to MAYV, ONNV, Ross River virus (RRV), and UNAV (Fig 3D). We found the highest cross-reactivity to ONNV and UNAV, with lower but readily detectable neutralizing antibody responses to MAYV, whereas neutralizing antibody responses to RRV were below the limit of detection (NT_50_<20). Similar neutralization patterns were observed with serum from animals immunized with a formaldehyde-based CHIKV vaccine, though their antiviral antibody responses were generally lower than that observed with the HydroVax-CHIKV approach (S3 Fig). A panel of naïve serum samples (*n* = 5) was also tested against each virus strain and found to be at or below the limit of detection. Together, these results indicate that HydroVax-CHIKV vaccination not only induces strong neutralizing antibody responses to heterologous CHIKV clades, but also elicits neutralizing antibody responses to at least three other closely related and clinically relevant alphaviruses.

### Antibody-mediated protection against CHIKV

**Fig 4:**
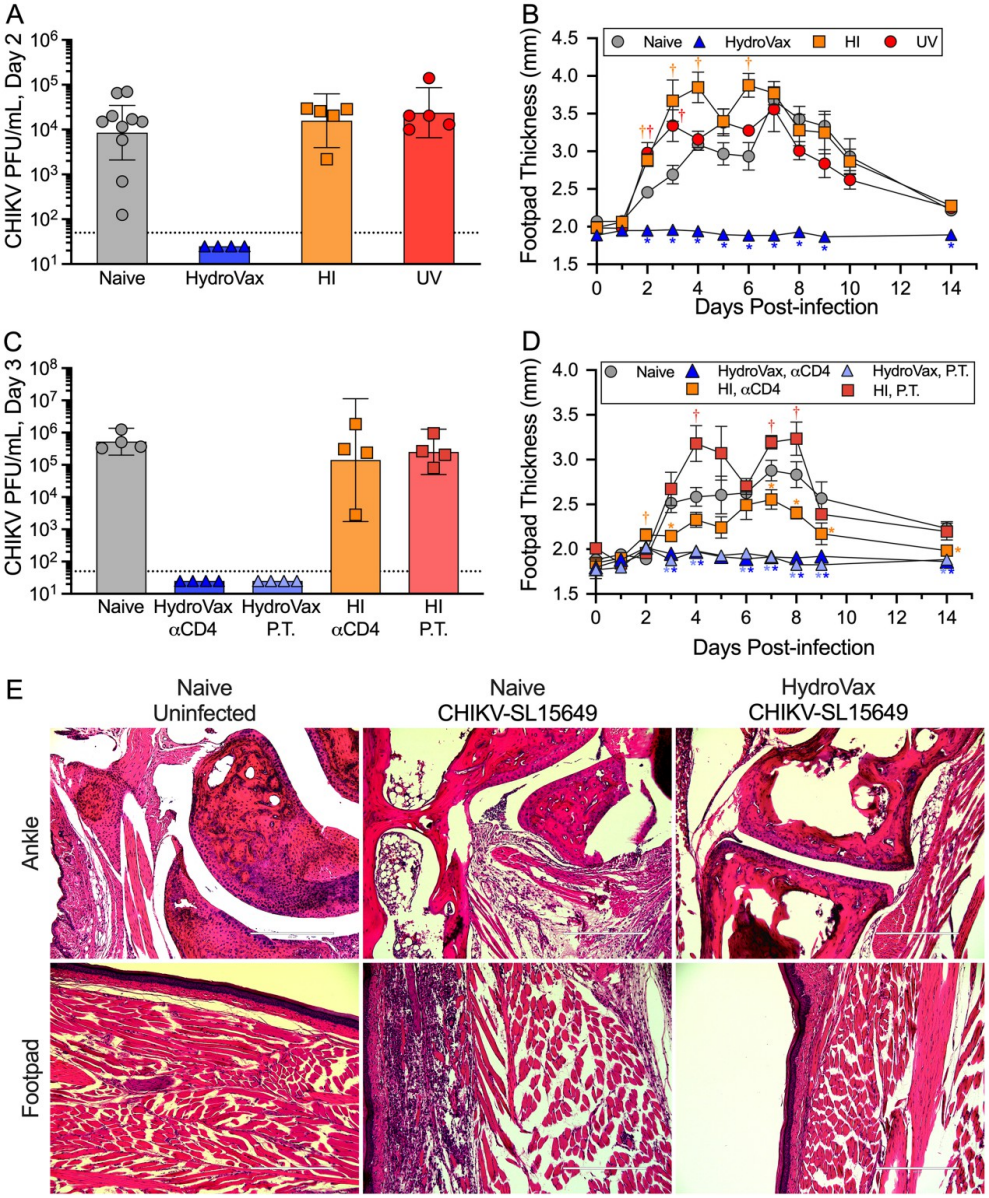
HydroVax-CHIKV vaccination protects against viremia and virus-associated arthritic disease following heterologous CHIKV challenge. Purified CHIKV-181/25 was inactivated with the optimized HydroVax approach (20 hours at room temperature), heat inactivation (HI-CHIKV, 1 hour at 80°C) or ultraviolet irradiation (UV, 10J). C57BL/6 mice (*n* = 4–10 mice per group) were immunized on days 0 and 28 with either a low dose of the optimized HydroVax-CHIKV (2 μg) or a high dose (5 μg) for HI-CHIKV and UV-CHIKV. At 3–4 months after primary immunization, mice were challenged in the footpad with 1,000 PFU of CHIKV-SL15649, along with age-matched naïve mice. (A) CHIKV viremia was assessed at 2 days post-challenge and (B) footpad swelling was monitored for up to two weeks. (C) Additional mice (*n* = 4 per group) were vaccinated with HydroVax-CHIKV or heat-damaged HI-CHIKV at 2 μg/dose on days 0 and 28 and challenged with CHIKV-SL15649 at 4 months post-primary vaccination. Animals from each group were depleted of CD4^+^ T cells or they received 300 μL of convalescent serum from HydroVax- or heat-inactivated vaccine-immunized animals at 1 day prior to challenge as indicated (passive transfer; P.T.) and viremia was measured by plaque assay on day 3 post-challenge. (D) Footpad swelling measurements were performed for 14 days after CHIKV-SL15649 challenge. For panels A and C, the dotted line indicates the limit of detection for viremia. Individual data points are shown along with the geometric mean represented by a bar and 95% confidence intervals. For panels B and D, group averages with ± SEM values are shown. Comparisons were made at each time point to naïve controls using ANOVA with multiple test correction. Significant differences (*P*<0.05) in footpad thickness relative to naïve mice are indicated, with a dagger symbol (†) signifying an increase in footpad swelling, while asterisks (*) denote a significant decrease in footpad swelling in comparison to naïve controls. (E) At 10 days post-infection, histopathology in the ankle joint (*upper panels*) and footpad (*lower panels*) was assessed among representative animals by hematoxylin and eosin staining; (*left*) naïve, uninfected mice; (*middle*) unvaccinated naïve mice after CHIKV infection; (*right*) HydroVax-CHIKV immune mice after CHIKV infection. White scale bars represent 400 μm.

A critical step in vaccine candidate selection is to assess protection/vaccine efficacy in a relevant model of human disease. We utilized a previously described challenge model in C57BL/6 mice in which animals are inoculated via the footpad with a heterologous ECSA clade clinical isolate, CHIKV-SL15649 [31]. Following footpad inoculation, animals demonstrate symptoms similar to human disease, including inoculation site inflammation, severe tenosynovitis and myositis, and high levels of viremia. Our immunogenicity results indicated that the HydroVax-CHIKV vaccine provided robust neutralizing antibody responses after immunization, whereas other inactivation approaches such as heat inactivation or UV treatment failed to elicit neutralizing antibody responses (Fig 3A) and in these studies we determined if vaccine-mediated protection could be attained in the absence of detectable neutralizing antibodies. Immunized mice were challenged with CHIKV-SL15649 at approximately 2–3 months after vaccination and assessed for viremia and footpad swelling to measure CHIKV-associated arthritic disease in comparison with unvaccinated controls. Naïve mice showed high levels of viremia by day 2 post-infection (GMT = 8.5x10^3^ PFU/mL; 95% CI: 2.1x10^3^–3.5x10^4^ PFU/mL) whereas HydroVax-CHIKV-immunized animals showed no detectable virus in circulation (<50 PFU/mL) (Fig 4A). In contrast, animals vaccinated with either heat-inactivated CHIKV or UV-inactivated CHIKV showed no protection against viremia with GMT = 1.6x10^4^ PFU/mL (95% CI: 3.9x10^3^–6.3x10^4^ PFU/mL) and 2.4x10^4^ PFU/mL (95% CI: 6.6x10^3^–8.6x10^4^ PFU/mL), respectively (Fig 4A). Viremia was undetectable in both vaccinated and unvaccinated animals at 7 days post-infection.

To measure protection against CHIKV-associated disease, footpad swelling was monitored over the course of 14 days after challenge and similar to prior studies [32], unvaccinated animals showed a rapid increase in footpad thickness that generally peaked by 7 days post-inoculation before returning close to baseline by day 14. Unlike naïve animals, HydroVax-CHIKV vaccinated mice demonstrated no signs of arthritic disease/footpad swelling throughout the 14-day period of observation (Fig 4B). Mice vaccinated with either heat-inactivated CHIKV or UV-inactivated CHIKV were not protected from CHIKV-associated disease and instead, were found to have exacerbated disease manifestations in comparison with unvaccinated mice (Fig 4B). Animals that had received either heat-inactivated CHIKV or UV-inactivated CHIKV vaccination demonstrated significantly increased footpad swelling in comparison to the unvaccinated, naïve controls as early as 2 days after challenge (*P*<0.05). To better understand the mechanisms underlying vaccine-mediated protection vs. exacerbated disease, further studies were performed to determine the role of vaccine-induced serum antibodies and CD4^+^ T cells. In these studies, CHIKV viremia titers among naïve animals reached 5.3x10^5^ PFU/mL (95% CI: 2.0x10^5^–1.4x10^6^ PFU/mL) at 3 days post-challenge (Fig 4C). In contrast, CD4^+^ T cell-depleted mice that had received HydroVax-CHIKV vaccination prior to challenge were still protected against viremia (<50 PFU/mL, Fig 4C) and from CHIKV-associated arthritic disease (Fig 4D), indicating that CD4^+^ T cells were not necessary for vaccine-mediated protection against CHIKV. Likewise, transfer of HydroVax-CHIKV immune serum resulted in complete protection against heterologous CHIKV viremia (<50 PFU/mL, Fig 4C) and CHIKV-associated arthritic disease (Fig 4D). This indicates that transfer of neutralizing antibodies alone was sufficient for protection against systemic spread of CHIKV and subsequent CHIKV-associated pathology. Mice vaccinated with heat-inactivated CHIKV showed high viremia similar to naïve controls (Fig 4A) and CD4^+^ T cell depletion of these mice likewise resulted in high viremia (GMT = 1.4x10^5^ PFU/mL; 95% CI: 1.8x10^3^–1.1x10^7^ PFU/mL) (Fig 4C). However, unlike the protection afforded by passive transfer of HydroVax-CHIKV immune serum, convalescent serum from HI-CHIKV immunized mice failed to provide protection against viremia (GMT = 2.5x10^5^ PFU/mL; 95% CI: 5.0x104–1.3x10^6^ PFU/mL). Passive transfer of immune serum from HI-CHIKV immunized mice did not alter the onset of arthritic disease at early time points after challenge (e.g., days 1–3), but footpad swelling was significantly increased (*P*<0.05) in these mice compared to naïve controls at 3/10 time points examined after challenge. CD4^+^ T cell depletion of HI-CHIKV immunized mice resulted in significantly reduced footpad swelling (*P*<0.05) compared to naïve controls at multiple time points after challenge, indicating that in the absence of a vaccine-induced neutralizing antibody response, vaccine-mediated exacerbation of CHIKV-associated disease could be at least partially mitigated by removal of CD4^+^ T cells. This result is consistent with prior studies indicating that CD4+ T cells are a major contributor to CHIKV-associated joint inflammation [32,33]. To determine if the absence of footpad swelling translated to vaccine-mediated protection against joint and muscle inflammation, the histopathology of these sites was examined (Fig 4E). Ankle and footpad tissues from representative uninfected naïve animals, unvaccinated (naïve) CHIKV-infected animals, and CHIKV-infected HydroVax-immunized animals were collected and stained with hematoxylin and eosin at 10 days post-infection, similar to prior studies [28]. Histological changes in musculoskeletal tissues were evaluated based on *(1)* inflammatory damage characterized by tendonitis, synovitis and arthritis in the ipsilateral talocrural joint (ankle) and *(2)* metatarsal muscle inflammation, edema and necrosis (footpad). CHIKV-infection of unvaccinated naïve mice demonstrated a marked distribution of lesions (51–75% of tissues) and clearly apparent inflammatory arthritis whereas HydroVax-CHIKV immunized animals had little to no inflammation (minimal distribution; 1–10% of tissues affected). These results are consistent with the lack of footpad swelling observed among vaccinated animals following CHIKV-SL15649 challenge (Fig 4B and 4D).

**Fig 5:**
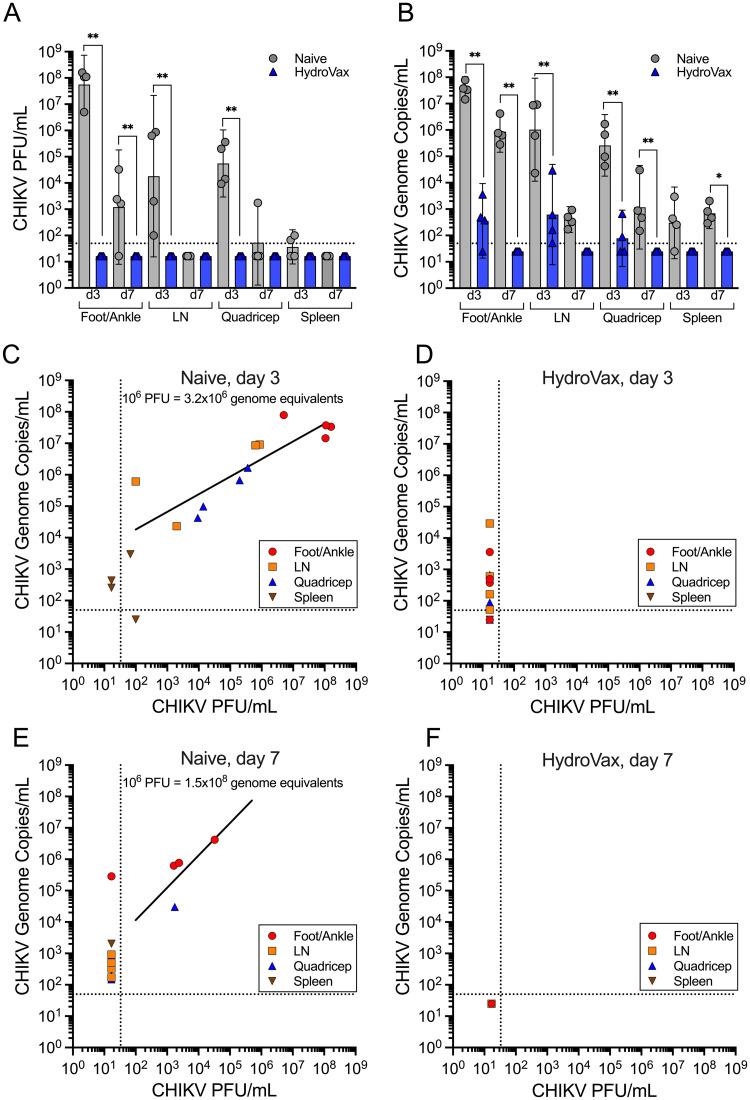
CHIKV dissemination is significantly reduced among HydroVax-immunized mice following heterologous challenge. Purified CHIKV-181/25 was inactivated with the optimized HydroVax approach (20 hours at room temperature) and formulated with alum adjuvant. C57BL/6 mice (*n* = 8 mice per group) were immunized at 2 μg per dose on days 0 and 28. At 56 days after primary immunization, mice were challenged in the footpad with 1,000 PFU of CHIKV-SL15649, along with age-matched naïve controls. At days 3 or 7 post-challenge, tissues from the challenge leg (foot/ankle, popliteal lymph node [LN] and quadricep muscle) and the spleen were collected from half of the animals in each group (*n* = 4 per timepoint). Tissues were homogenized and assayed directly for (A) CHIKV virus titers or (B) further processed for RNA and assayed for CHIKV genome equivalents. RNA levels were normalized to the relative expression level of ribosomal protein S17 (RPS17). Pairwise comparisons were made between naïve and vaccinated groups at each time point in each tissue set using ANOVA with multiple test correction (*, *P*<0.05; **, *P*<0.01). Comparisons between genome copies and infectious titers (PFU/mL) in harvested tissues were performed at both day 3 (C, D) and day 7 (E, F) for naïve and HydroVax-CHIKV vaccinated animals. Dotted lines represent limits of detection.

To further characterize HydroVax-CHIKV vaccine-mediated protection, we measured viral dissemination in multiple tissues including the foot/ankle, draining popliteal lymph node (LN), quadricep muscle, and spleen at 3 and 7 days post-infection. Infectious virus was detected in all tissues from naïve mice at day 3 post-infection, with the exception of the spleen in which infectious virus was detected in only 2/4 animals (Fig 5A). By day 7 post-infection, CHIKV titers had declined from the peak on day 3 but were still detectable in footpad/ankle samples in 3/4 naïve animals. In stark contrast, no infectious virus was detected from vaccinated animals in any of the tissues examined at either day 3 or day 7 post-infection. This difference was most striking in foot/ankle samples at day 3 post-infection, with CHIKV titers reaching 5.6x10^7^ PFU/mL in naïve animals, compared to a greater than one million-fold reduction of virus titers to below the limits of detection (< 33 PFU/mL) among HydroVax-CHIKV recipients. Significant differences between vaccinated and unvaccinated groups of animals were also observed using quantitative RT-PCR-based detection of CHIKV genome copy numbers (Fig 5B). CHIKV genome copies were highest at day 3 post-infection among naïve animals and remained detectable in all tissues at day 7 post-infection. In contrast, although residual CHIKV RNA was detected in some tissue samples from vaccinated animals at day 3, CHIKV RNA was undetectable (< 50 copies/mL) by day 7 post-infection in all tissues (Fig 5B). CHIKV infectious virus titers were similar to CHIKV RNA genome copy numbers, at the day 3 timepoint in naïve animals (1x10^6^ PFU = 3.2x10^6^ genome copies; Fig 5C), whereas this association could not be determined when examining vaccinated animals due to the absence of detectable live virus at this early time point (Fig 5D). Interestingly, this 1:3 ratio of infectious virus particles to virus genome equivalents at day 3 shifted to a ratio of 1:150 infectious virus particles to virus genome copies by day 7 post-infection in tissues where live virus could still be detected (1x10^6^ PFU = 1.5x10^8^ genome equivalents; Fig 5E). This may be due to the adaptive immune response in the naïve animals eliminating infectious virus while nonviable viral RNA remains at least transiently detectable. Similar correlation analysis was not possible with the HydroVax-CHIKV animals since both infectious CHIKV and CHIKV RNA were below the limits of detection at 7 days post-infection (Fig 5F).

**Fig 6:**
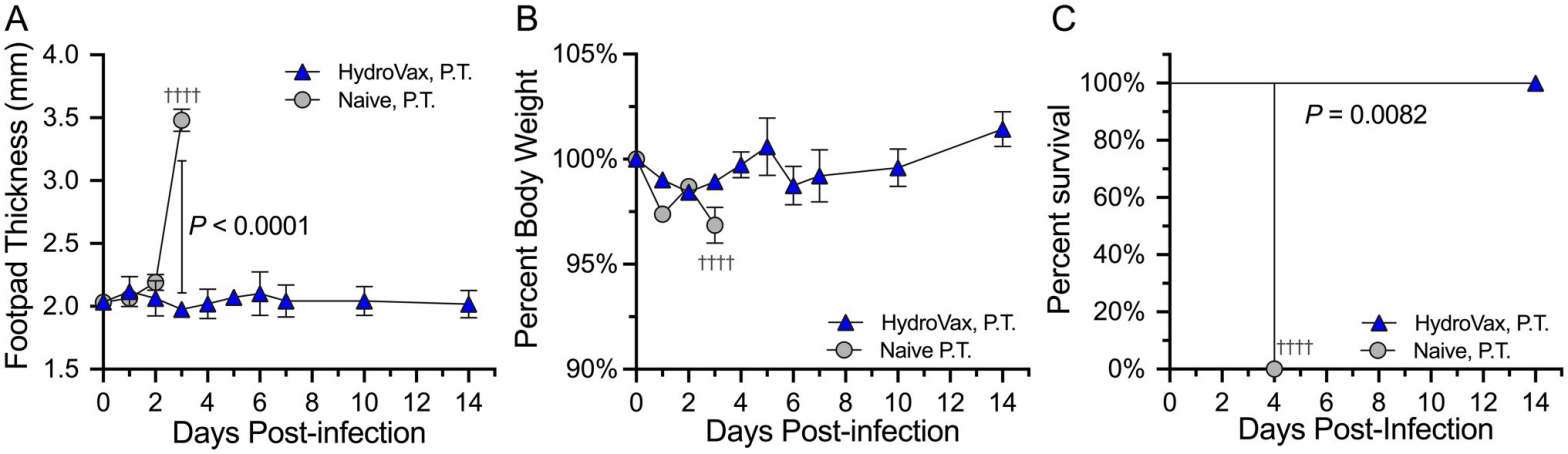
Vaccine-induced serum antibodies protect against lethal CHIKV challenge. Pooled immune sera from HydroVax-CHIKV vaccinated C57BL/6 mice, or matched naïve controls, was administered to AG129 mice (*n* = 4 per group) one day prior to heterologous footpad challenge with 1,000 PFU of CHIKV-SL15649. Group average footpad thickness values (A) or change in weight (B) are provided with ± SEM. Significant differences in footpad swelling at 3 days post-infection were determined using unpaired Student’s *t*-test. (C) Group survival comparisons were performed using the Mantel-Cox log rank test. Dagger symbols represent mortality events. P.T; passive transfer.

In addition to virus-induced arthritic disease, severe CHIKV can result in a lethal infection in some cases [4–6]. AG129 mice, which lack the IFN-α/β (type I interferon) and IFN-γ (type II interferon) receptors, are highly susceptible to viral pathogens including CHIKV [34]. To determine if HydroVax-CHIKV vaccine-induced antibodies can protect against lethal CHIKV challenge, we administered either HydroVax-CHIKV immune serum, or naïve serum from unvaccinated control animals to adult AG129 mice one day prior to challenge. Mice were infected with 1,000 PFU of CHIKV-SL15649 in the footpad and monitored for changes in footpad thickness, weight, and mortality for up to 14 days (Fig 6). Animals that received naïve serum showed rapid and significant footpad swelling between day 2 and day 3 post-infection whereas animals that received HydroVax-CHIKV immune serum showed no footpad swelling at any time point (Fig 6A). Mice treated with naïve serum lost <5% of their weight after CHIKV infection whereas mice that received HydroVax-CHIKV immune serum maintained stable weight that slowly increased over the following 14 days of observation (Fig 6B). All of the AG129 mice that received naïve serum succumbed to CHIKV at 4 days post-infection whereas animals that received HydroVax-CHIKV immune serum demonstrated 100% survival (*P* = 0.0082; Fig 6C). In total, these studies indicate that HydroVax-CHIKV vaccine-induced serum antibodies are sufficient for protection against both acute (Fig 4) and lethal (Fig 6) CHIKV infection.

## Discussion

Chikungunya virus (CHIKV) is an alphavirus that has spread globally due to the distribution and abundance of its mosquito vectors, *Aedes aegypti* and *Aedes albopictus* [1]. CHIKV infection may result in a febrile illness characterized by an intensely painful polyarthralgia, with the potential for debilitating long-term sequelae and in severe cases, death [3–6,35]. Using an advanced site-directed oxidation-based inactivation approach, we developed a new CHIKV vaccine, HydroVax-CHIKV, that efficiently maintains neutralizing epitopes after virus inactivation, induces broad neutralizing antibody responses against representative strains of the 4 CHIKV clades as well as other related alphaviruses (MAYV, ONNV, UNAV), and provides complete antibody-mediated protection against viremia, arthritic disease and lethality following heterologous challenge with a virulent clinical isolate, CHIKV-SL15649. Together, these findings support the further development of HydroVax-CHIKV as a novel vaccine candidate to address this unmet clinical need.

CHIKV was first described in modern-day Tanzania during the 1950s, with subsequent sporadic outbreaks recorded in Africa and Southeast Asia, followed by a period of inactivity from the 1980s through the turn of the 20^th^ century [35]. However, this pattern changed dramatically starting with a 2004 outbreak on the Kenyan coast, leading to more than a decade of global expansion [35]. Following its reemergence, an estimated 1.3 million infections occurred in India during the 2006–2007 epidemic [36] and >2.9 million cases in the Caribbean and the Americas from 2013–2016 [2]. Based on the broad global spread of CHIKV, over 1 billion people remain at risk for infection. CHIKV variants have been found to readily infect the mosquito vector *Aedes albopictus*, which is widespread in various countries across Europe as well as the Southeastern and Midwestern United States, indicating there is risk that the virus could become endemic across an even broader geographical range [3]. International travelers leaving endemic regions can be viremic and transport CHIKV back to their country of origin. For instance, transmission originating from an international traveler resulted in an estimated 205 locally acquired autochthonous CHIKV cases in Italy in 2007, demonstrating that this is not just a theoretical concern [37]. Despite the wide geographical distribution of CHIKV and its potential for further spread, there is currently no commercial vaccine available to combat this disease.

Passive administration of immunoglobulins, from either convalescent cases or as monoclonal antibodies, has demonstrated protection against CHIKV disease in several animal models including mice [28] and rhesus monkeys [38]. Accordingly, CHIKV-specific neutralizing antibodies have been proposed as the primary correlate of protection against CHIKV infection, specifically in the context of prophylactic vaccine development [39]. A number of preclinical CHIKV vaccine candidates have been described including CHIKV inactivated with formaldehyde, BPL, tween-ether extraction and UV irradiation [35], in addition to DNA vaccines, protein subunit vaccines, virus-like-particles (VLP), recombinant/chimeric candidates, and live-attenuated CHIKV vaccines [35] with variable results. For example, a CHIKV vaccine based on the Modified vaccinia Ankara (MVA) expression system was tested in a two-dose series (days 0 and 28) in mice, but animals reached an average neutralization titer of NT_50_ = 9.2 at two weeks post-boost and passive antibody transfer showed no protection against disease [40]. CHIKV vaccines based on either formaldehyde or BPL, have also been described [41]. In those studies, a 10 μg dose of formaldehyde- or BPL-based vaccine, given in a 2-dose series (days 0 and 14), elicited neutralizing GMTs of 320 and 640, respectively at two-weeks post-boost, increasing to GMTs of only 1280 and 2560 when using a dose as high as 50 μg. In that study, the use of a recombinant E2 protein-based subunit vaccine was also shown to have limited immunogenicity, eliciting a GMT of 320 after administration of a 50 μg dose in a two-dose series [41]. Development of a CHIKV vaccine using an advanced VLP system provided higher immunogenicity in mice, in which a two-dose vaccination series (days 14 and 42), using 19 μg of purified E1/E2 VLP combined with the oil-in-water Ribi adjuvant, elicited homologous GMTs of 10,703 at 10 days after booster vaccination [42]. By comparison, following a two-dose vaccination schedule with 0.5 μg of HydroVax-CHIKV, mice reached homologous GMTs of 20,400, despite the use of a nearly 40-fold lower dose of vaccine antigen relative to the CHIKV E1/E2-VLP vaccination studies. In total, comparison of preclinical data indicates that HydroVax-CHIKV is highly immunogenic relative to other potential vaccine candidates.

At least six vaccine approaches have been reported in human clinical studies [43]. One of the earliest clinical vaccine candidates was a formaldehyde-inactivated CHIKV vaccine developed in 1971, which was described as safe and immunogenic in a small number of human subjects [44] but was not pursued further, due to vaccine potency issues [45]. A live-attenuated vaccine, TSI-GSD-218 (CHIKV-181/25), was found to be immunogenic in Phase 1 and Phase 2 trials, but approximately 8% of the vaccinees developed transient arthralgia and further vaccine development was halted due to these reactogenicity concerns [45]. The live-attenuated CHIKV vaccine, VLA1553, incorporates a 60 amino acid deletion in the nsP3 protein and although it induces neutralizing antibodies and recorded no serious adverse events in Phase 1 clinical trials, the vaccine is associated with some systemic reactogenicity [46] with moderate to severe fever observed in 9.7%, 13.3% and 32.2% of participants in the low-, medium-, and high-dose groups following primary vaccination, respectively. A measles virus-based recombinant CHIKV vaccine, MV-CHIK, demonstrated peak GMT = 175 at four weeks post-booster vaccination following a two-dose schedule (days 0 and 28), but antibody responses declined to GMT = 39 within 5 months [47]. The VRC-CHKVLP059-00-VP virus-like particle (VLP) has been recently tested in a Phase 2 clinical trial [48]. The study investigated a 20 μg dose level using a 2-dose series administered at 0 and 4 weeks in an endemic setting [48]. Among baseline seronegative subjects (GMT = 15.7) neutralizing antibody titers increased to GMT = 119 by week 4 and were boosted to GMT = 1728 by week 8. However, by 72 weeks after vaccination the antiviral antibody titers had declined to GMT = 98. An mRNA-based CHIKV vaccine has also been described [43] but published descriptions have been limited to a meeting abstract with few details at this time [49]. In total, despite significant efforts in the field, new and improved CHIKV vaccines are needed to protect at-risk populations against this widespread mosquito-borne disease.

The use of H_2_O_2_ alone for the development of inactivated whole-virus vaccine candidates has shown protective efficacy across a number of animal models [8–14]. However, we have recently developed an advanced, site-directed oxidation approach which allows for the use of substantially reduced concentrations of H_2_O_2_ during inactivation while still providing rapid inactivation kinetics and the maintenance of antigenic structures [15,16]. This is achieved through the use of the redox-active metal copper (Cu) complexed with the antiviral compound, methisazone (MZ), which together have an affinity for nucleic acids [21]. Although the precise mechanism for virus inactivation is not fully defined, the ability of Cu, in the presence of H_2_O_2_, to induce DNA and RNA breaks as well as individual base damage is well-established and is postulated to operate through site-specific binding of Cu to nucleic acid, followed by the localized production of hydroxy radicals [50,51]. The H_2_O_2_-based inactivation observed in the presence of MZ-Cu complexes is not only rapid, but also results in less damage to neutralizing epitopes and is consistent with the theory that this complex specifically targets nucleic acids, with localized oxidation leading to single and double stranded breaks while limiting free Cu from participating in oxidative damage to amino acids in neutralizing epitopes. During the optimization of this platform technology for CHIKV vaccine development, the combination of H_2_O_2_ (0.0003%) and CuCl_2_ (2 μM) resulted in relatively slow inactivation kinetics (H_2_O_2_/ CuCl_2_; T_1/2_ = 96 min), but this rate increased dramatically (36-fold) with the addition of MZ (H_2_O_2_/CuCl_2_/MZ; T_1/2_ = 2.7 minutes), while effectively protecting neutralizing epitopes from the severe oxidative damage that is observed after exposure to 3% H_2_O_2_ alone (S1 Fig). The ability of this inactivation approach to specifically target viral nucleic acid is further supported by Northern blot data demonstrating the rapid degradation of genomic CHIKV RNA (Fig 2C), which coincides with a rapid loss of infectious virus (Fig 2B) while neutralizing protein epitopes remain intact during the course of inactivation (Fig 2D).

Maintenance of antigenic structure is a key aspect of vaccine development and the approach used to prepare inactivated vaccines can greatly alter experimental results as well as have real-world consequences [52]. For instance, during the development of an Ebola vaccine candidate, an independent group compared γ-irradiation to H_2_O_2_-based inactivation [13]. The vaccine candidate treated with γ-irradiation provided no protection against viremia in non-human primates (NHP) and animals were fully susceptible to lethal Ebola challenge. In contrast, a similar vaccine treated with H_2_O_2_ provided complete protection against viremia and the H_2_O_2_-Ebola immunized animals survived a normally lethal dose of Ebola. The difference between these two inactivation approaches is likely due to the damage or maintenance of protective epitopes. From a real-world perspective, formaldehyde-based vaccines against respiratory syncytial virus (RSV) and measles virus provide examples in which vaccination with a damaged vaccine antigen may result in poor protection or possibly even exacerbated disease due to vaccine-associated immunopathology [52]. For CHIKV, we examined a number of physical and chemical approaches to inactivate live virus (Fig 2A and 2B), but in some cases (e.g., heat-, UV-, and BPL-based inactivation) this led to extensive antigenic damage (Fig 2D) and a dramatic impact on vaccine efficacy, compromising both the ability to induce functional neutralizing antibody responses (Fig 3) and to protect against CHIKV-associated disease (Fig 4). Indeed, vaccination of mice with heat-inactivated CHIKV or UV-irradiated CHIKV not only resulted in loss of protection against viremia, but also significantly increased CHIKV-associated arthritic disease at early time points after challenge, a result that could be significantly reduced by depleting CD4^+^ T cells (Fig 4). Similar results have been observed with experimental CHIKV vaccines based on recombinant cytomegalovirus or adenovirus vectors that induced antiviral T cell responses in the absence of CHIKV-specific neutralizing antibodies [32]. These T cell-based vaccines did not protect against CHIKV viremia and likewise were associated with significantly increased footpad swelling after CHIKV challenge. Interestingly, the T cell-based immunopathology associated with these vaccines may have been due to induction of CHIKV-specific CD4^+^ T cells since incorporation of only CHIKV-specific CD8^+^ T cell epitopes into recombinant adenovirus vectors resulted in reduced footpad swelling after CHIKV challenge instead of exacerbation of virally-induced arthritic disease [32]. Other examples of vaccine-associated immunopathology have been identified in pigs and ferrets vaccinated with mismatched UV-inactivated influenza virus that was unable to elicit a cross-neutralizing antibody response prior to viral challenge [53,54]. It is believed that this form of immunopathology during influenza infection may be due to an imbalance of pre-existing T cell-mediated immunity in the absence of a protective neutralizing antibody response [55]. The risks associated with vaccine-induced CD4^+^ T cell memory in the absence of protective antiviral antibody responses was also elegantly demonstrated in a mouse model of chronic lymphocytic choriomeningitis virus (LCMV) infection [56]. In this study, prior vaccination of mice with a potent LCMV-specific CD4^+^ T cell epitope vaccine resulted in “catastrophic” immunopathology and unanticipated mortality after chronic LCMV challenge. However, vaccine-induced CD4^+^ T cell-mediated immunopathology could be ameliorated by co-transfer of an LCMV-specific neutralizing monoclonal antibody to vaccinated mice prior to viral infection. Although CD4^+^ T cell help is critical for the induction of optimal antiviral CD8^+^ T cell and antibody responses, these results indicate that unbalanced vaccine-induced T cell-mediated immunity in the absence of a protective neutralizing antibody response may be potentially harmful to the host and illustrates the importance of developing appropriate animal models for selecting vaccines for future clinical development.

CHIKV remains a viral pathogen of global concern, with the capacity for explosive epidemics that can result in debilitating and long-lasting arthritic disease [3]. In severe cases, CHIKV infection can lead to death, with case fatality rates (CFR) ranging from approximately 1 death per 1000 to 8000 reported cases [4,5]. However, this range may represent an underestimate of the true burden of disease. Frutuoso et al reported an estimate of approximately 0.7 deaths per 1000 cases based on data reported by the Brazilian Health Ministry [6]. However, the authors contended that the Brazilian health care system at that time was not able to fully and accurately detect and record severe CHIKV cases and deaths, resulting in a systematic underestimation of CHIKV-related mortalities. The authors reported a corrected CFR, with a window of observation that extended out to 6 months following symptom onset and estimated a lethality rate of 5.7 deaths per 1000 cases of CHIKV (i.e., 1 death per 175 cases) [6].

Other arthritogenic alphaviruses including MAYV, ONNV, and UNAV cause substantial acute and chronic human disease and contain cross-reactive neutralizing epitopes [57]. In our studies, HydroVax-CHIKV vaccination elicited substantial cross-neutralization against other alphaviruses (Fig 3D), albeit at lower titers compared to CHIKV itself, which is not unexpected since these are genetically distinct alphaviruses. In general, the patterns of cross-neutralization track with the phylogenetic relatedness of these virus strains. CHIKV, ONNV, MAYV, UNAV and RRV are all part of the Semliki Forest virus (SFV) alphavirus complex, but vary in their level of relatedness to each other. The E2 protein is considered the primary target of neutralizing antibodies, and CHIKV and ONNV show >90% amino acid similarity in the E2 protein [58], which agrees with the high level of cross-neutralization that we observed (Fig 3D). Cross-neutralization from naturally infected CHIKV human cases against MAYV and UNAV has been previously reported [58]. Similar to our results, serum samples from CHIKV-infected individuals developed neutralizing antibody titers that were typically higher against UNAV compared to MAYV. This trend cannot be strictly explained by overall phylogenetic relatedness, as both UNAV and MAYV are closely related to each other, and a similar phylogenetic distance from CHIKV. Based on the antibody profiles that we observed in our studies (Fig 3D), the immunodominant neutralizing antibody epitopes from CHIKV may be more closely conserved with UNAV than to MAYV. Likewise, while the phylogenetic relatedness of RRV to CHIKV is similar to the distance observed for UNAV or MAYV [58], it appears that the dominant antibody responses elicited by CHIKV vaccination are unable to efficiently neutralize RRV.

Despite the substantial and continuing risk posed by CHIKV and other medically important alphaviruses, the goal of a licensed vaccine against any one of these viruses remains unmet. Here, we have developed a next-generation inactivated CHIKV vaccine based on an advanced, site-directed oxidation technology. Using this approach, we identified a vaccine candidate that preserved the antigenic structure of CHIKV and induced high-titer neutralizing antibodies against heterologous CHIKV strains from the 4 major genotypes in addition to other closely related alphaviruses of clinical importance including MAYV, ONNV, and UNAV. HydroVax-CHIKV vaccination provided antibody-mediated protection against viremia, arthritic disease and lethal CHIKV infection. Prior studies have indicated that CHIKV-immune mice are protected against MAYV, ONNV and Semliki Forest virus (SFV) [59–61], indicating that there is potential for the HydroVax-CHIKV vaccine to provide broader antiviral immunity to other arthritogenic alphaviruses in addition to CHIKV itself and these studies deserve further investigation. Together, we believe that the results provided here support the continued development of HydroVax-CHIKV as a new approach for protecting at-risk populations against these persistent public health threats.

## Materials and methods

### Ethics statement

All animal studies were approved by the OHSU Institutional Animal Care and Use Committee (IACUC) in accordance with the National Institutes of Health guide for the care and use of laboratory animals, protocol number TR01_IP00000801. Animals were humanely euthanized by the overdose administration of inhalant anesthesia (isoflurane). This method is considered acceptable according to AVMA Guidelines for the Euthanasia of Animals.

### Cells and viruses

CHIKV-181/25, CHIKV-37997 and CHIKV-La Reunion (CHIKV-LR, strain 2006-OPY1) were generously provided by Dr. Robert Tesh (World Reference Center for Emerging Viruses and Arboviruses [WRCEVA], University of Texas Medical Branch, Galveston, TX). CHIKV-SL15649 was derived from an infectious cDNA clone [31] generously provided by Dr. Mark T. Heise (University of North Carolina at Chapel Hill). ONNV-UgMP-30 (NR-51661), MAYV-BeAr505411 (NR-49910), RRV-T48 (NR-51457), and UNAV-MAC-150 (NR-49912) were obtained from BEI Resources, NIAID, NIH. MAYV, ONNV, RRV and UNAV were amplified on C6/36 cells and titered on Vero cells. CHIKV-181/25 was expanded through three passages on serum-free Vero cells and then sequentially triple-plaque-purified to ensure clonality on serum-free adapted Vero cells during development of a pre-master virus bank (pre-MVB). This pre-MVB was propagated under cGMP compliance to prepare a CHIKV-181/25 master virus bank (MVB), and the MVB was subsequently Sanger sequenced (Genewiz, Inc., South Plainfield, NJ).

### Virus plaque assays

Plaque assays were performed similar to prior descriptions [8]. Briefly, CHIKV samples were 10-fold serially diluted in growth medium (DMEM supplemented with 10% fetal bovine serum [FBS]) and dispensed at 0.2 mL per well onto confluent Vero cell monolayers in 6-well plates. Following a 1 hr incubation at 37°C/5% CO_2_, the wells were overlaid with 3 mL of 0.5% agarose in EMEM containing 2.5% FBS, 2 mM glutamine and antibiotics and incubated for 1 day at 37°C/5% CO_2_. Plates were removed from the incubator and each well was then overlaid with 1 mL of a 1% agar solution containing 0.015% (w/v) neutral red stain followed by overnight incubation at 37°C/5% CO_2_. Plaque assays for MAYV, ONNV, RRV and UNAV were performed similarly with the following modifications. Virus dilutions were inoculated onto 12-well plates of confluent Vero cells, and incubated for 2 hours at 37°C. The wells were overlaid with a 2:1 mixture of DMEM (supplemented with 5% FBS) and 2% carboxymethylcellulose. UNAV, MAYV, and RRV plates were incubated for two days and ONNV plates were incubated for three days at 37°C/5% CO_2_. Plates were fixed with 3.7% formalin and plaques were visualized with 0.2% methylene blue. For virus inactivation studies, samples were removed at pre-specified timepoints and chemical inactivation agents were rapidly diluted prior to assessment of live virus titers by plaque assay.

### Gel electrophoresis and mass spectrometry

Reducing SDS-PAGE was performed with NuPAGE 4–12% Bis-Tris gels in MOPs running buffer per the manufacturer’s directions (ThermoFisher Scientific). Briefly, samples were diluted into NuPAGE LDS sample buffer supplemented with a final concentration of 50 mM dithiothreitol, heated at 80°C for 10 minutes, and loaded onto prepared gels. Gels were run in an Xcell SureLock System (ThermoFisher Scientific) for approximately 45 minutes at 200V and stained with the Pierce Silver Stain Kit according to manufacturer’s directions (ThermoFisher Scientific). For protein band evaluation, major bands were excised and analyzed by mass spectrometry following trypsin digestion (MS Bioworks, Ann Arbor, MI).

### Vaccine production

CHIKV-181/25 was propagated on serum-free Vero cells, with harvests clarified and treated with Benzonase to minimize host-cell DNA/RNA contamination prior to concentration and buffer-exchange using tangential flow filtration (TFF) followed by CaptoCore 700 chromatography (Cytiva). All vaccine approaches utilized the same high-purity live CHIKV-181/25 material as their starting point. HydroVax-based inactivation conditions were optimized for CHIKV-181/25 and included 0.0003% H_2_O_2_, 2 μM CuCl_2_, 20 μM MZ and 0.06% formaldehyde, in a buffer matrix containing a protective level of polyatomic oxyanions [22] (150 mM Na_2_HPO_4_) at pH = 7.5, for 20 hours at room temperature. Other inactivation approaches included heat inactivation (HI; 1 hour at 80°C), ultraviolet irradiation (UV, 10 Joules at room temperature using a Spectrolinker XL-1000 UV crosslinker), β-propiolactone (BPL, 0.10% for 20 hours at room temperature, supplemented with addition of 50 mM HEPES), and formaldehyde (0.01% for 20 days at 37°C as previously described [24]). Following inactivation, chemical components were removed using ion-exchange chromatography, TFF or dialysis with complete inactivation confirmed through cell culture-based residual live virus testing. Vaccine antigens were formulated with 0.2% aluminum hydroxide (Alhydrogel, InvivoGen) prior to use.

### CHIKV ELISA

To assess antigenic changes during the inactivation process, both capture and indirect CHIKV-specific ELISA approaches were used. For capture ELISA, a CHIKV-specific assay was developed, similar to previous descriptions for an inactivated West Nile virus (WNV) vaccine candidate [10]. This approach consisted of a sandwich ELISA using a CHIKV-specific, neutralizing monoclonal antibody (MAb) designated clone 13b8 (CHIKV NT_50_ = 4 ng/mL), which was shown to target the E2 envelope protein as determined by recombinant protein binding studies. The CHIKV neutralizing MAb 13b8 was derived from a BALB/c mouse, infected with CHIKV-181/25 with hybridoma fusion to the P3X63Ag8.653 murine myeloma cell line performed according to standard protocols [62]. For capture ELISA, MAb 13b8 (1 μg/mL) was used to coat ELISA plates (Polystyrene High Bind, Corning) overnight at 2–8°C. Unbound antibody was removed, and plates were treated with blocking buffer (5% non-fat dry milk in PBS-T [PBS supplemented with 0.05% Tween-20]) for 1 hour at room temperature. Plates were rinsed with PBS-T and incubated for 1 hour with live, untreated control CHIKV antigen, or inactivated CHIKV antigens, at a concentration of 1 μg/mL. Plates were washed with PBS-T and incubated with biotinylated MAb 13b8 (0.5 μg/mL) for 1 hour at room temperature. Plates were rinsed again with PBS-T and incubated with an optimal dilution of streptavidin poly-HRP (ThermoFisher Scientific). After a final wash, plates were developed with o-phenylenediamine dihydrochloride (OPD) substrate in citrate buffer for 15 minutes, with development stopped by addition of an equal volume of 1M HCl, and optical densities (OD) were measured at 490 nm. ODs were blank-subtracted, and the percentage of live virus signal was calculated for each inactivation condition by comparison to the matched live virus control. Indirect ELISAs were performed using the previously described neutralizing CHK-152 and CHK-166 MAbs [28], generously provided by Dr. Michael S. Diamond (Washington University-St. Louis), which map to distinct epitopes on the E1 (CHK-166) or E2 (CHK-152) proteins of the CHIKV envelope, as well as the 13b8 MAb. For these assays, purified live or inactivated virus was diluted to 1 μg/mL in PBS and used to coat ELISA plates overnight at 2–8°C. Unbound antigen was removed, and plates were treated with blocking buffer as above. Plates were rinsed with PBS-T and incubated for 1 hour with antibody diluted in PBS (0.25 μg/mL). Plates were washed with PBS-T and incubated with an optimal dilution of goat anti-mouse IgG-HRP (ThermoFisher Scientific) and ELISA plates were developed as described above.

### CHIKV genomic RNA analysis by Northern blotting

Purified CHIKV-181/25 was inactivated using the HydroVax-based platform and samples were removed at selected time points, with inactivation components neutralized using a final concentration of 1 mM ethylenediaminetetraacetic acid (EDTA) and 1 mM sodium metabisulfite, followed by a hold step on ice. Genomic CHIKV RNA was extracted with TRIzol using 3 μl of linear acrylamide as a carrier molecule, and Northern blotting was performed as previously described [27]. Briefly, equal volumes of extracted RNA were electrophoretically separated on a 1.2% formaldehyde agarose gel and transferred onto a Nylon positively charged membrane (Roche). CHIKV RNA was detected using an E2-6K-E1-specific digoxigenin (DIG) probe (Roche) constructed by PCR using forward primer 5’-CGCAGTTATCTACAAACGGTA-3’ and reverse primer 5’-TTTACTCTCAGGTGTGCGA-3’. Detection was performed using the DIG-High Prime DNA labeling and detection starter kit II (Roche). DIG-labeled membranes were incubated with the CSPD chemiluminescent substrate and visualized on CL-XPosure film (ThermoFisher Scientific).

### Histological analysis

For histological analysis, mice were euthanized and perfused by intracardial injection of 4% paraformaldehyde (PFA). Hind limb tissues from infected legs were removed and fixed in 4% PFA and embedded in paraffin with 5 μm sections prepared, stained with H&E and evaluated by light microscopy (Olympus VS120 Virtual Slide Microscope). Anatomic pathology specialists scored the presence, distribution and severity of histological lesions, using a scoring system as follows: Scoring 0–5: 0 absent (no lesions), 1 minimal (1~10% of tissues affected), 2 mild (11~25% affected), 3 moderate (26~50% affected), 4 marked (51~75% affected), 5 severe (>75% affected).

### Tissue viral loads

Tissues were homogenized in 800 μL of 1X PBS plus 200 μL of solid glass beads (Propper) using a bead beater system (Precellys 24 homogenizer, Bertin Technologies), and homogenates were cleared of cellular debris through centrifugation (5,000×*g* for 2 min). Infectious virus titers were determined by transferring 20 μL of tissue homogenate to 180 μL DMEM supplemented with 5% FBS and completing a total of six 1:10 serial dilutions. Following dilution, 100 μL of diluted homogenate was transferred to confluent monolayers of Vero cells. Triplicate plaque assays were performed as described above with a 2-day incubation period. CHIKV genome equivalents were determined by Real Time PCR detection of a sequence specific to CHIKV E1 with forward primer 5’-GAGGTGTGGGACTGGTTGTTG-3’, reverse primer 5’-CAAGTTAGTGCCTGCTGAACGA-3’ and probe 5’-FAM-AATCGTGGTGCTATGCGT-3’-MGB. Total RNA was extracted from 200 μL of clarified tissue homogenate using Direct-zol RNA isolation kit (Zymo Research) following the manufacturer’s protocol. cDNA was generated from 1 μg of DNAse-treated total RNA with Superscript IV reverse transcriptase (ThermoFisher Scientific) following the manufacturer’s protocol. Normalization of sample input was performed utilizing the housekeeping gene, Rps17, forward primer 5’-GCCATTATCCCCAGCAAGAA-3’, reverse primer 5’-CTTCATCAGATGCGTGACATAGC-3’ and probe 5’-FAM-CTTCGGAACAAGATAGC-3’-MGB. Data analysis was performed using QuantStudio Real-Time PCR software.

### Serum ELISA and neutralization (NT_50_) assays

Total CHIKV-binding serum IgG was assessed by ELISA using an optimized concentration of live CHIKV-infected Vero cell lysate similar to previous studies involving other viruses [15]. Serum neutralization titers-50% (NT_50_) were determined using a plaque reduction assay by incubating 10-fold serial dilutions of heat-inactivated mouse serum with approximately 40–100 PFU of each virus for 2 hours at 37°C prior to plating the virus on confluent Vero cell monolayers and performing plaque assays as described above. The NT_50_ titer was defined as the last serum dilution in which at least 50% of input virus was neutralized.

### Animals

Female BALB/c ByJ (monoclonal antibody development) or C57BL/6 (vaccination studies) mice were obtained from the Jackson Laboratory (Bar Harbor, ME). Primary vaccinations were administered by the intraperitoneal route between 7–9 weeks of age. AG129 mice were bred and housed at OHSU and used at 17 weeks of age. For CHIKV challenge studies, mice were inoculated via the footpad route with 1,000 PFU of CHIKV-SL15649 as described [32]. Where indicated, CD4^+^ T-cell depletion was performed via intraperitoneal (i.p.) administration of MAb GK1.5 (αCD4 rat IgG; 150 μg per dose) on days -1, 0 and 2 relative to challenge. A 3-dose series using at least 100 μg per dose of GK1.5 results in >90% CD4^+^ T-cell depletion (S4 Fig). Our prior studies have also demonstrated that administration of rat IgG isotype control does not impact CHIKV infection or pathogenesis [32]. For passive serum transfer studies into C57BL/6 mice, donor animals were vaccinated with 2 μg of HydroVax-CHIKV or heat-inactivated CHIKV on days 0 and 28, with serum collected and pooled at day 56 post-vaccination. For passive transfer studies in AG129 mice, serum was collected and pooled from HydroVax-CHIKV vaccinated C57BL/6 animals (2 μg on days 0 and 28) at days 35, 42 and 56 post-vaccination, or serum was pooled from matched naïve controls. All recipient animals received 300 μL of pooled immune serum at 1 day prior to challenge.

### Statistics and inactivation kinetic modeling

Inactivation kinetics were fit using a least squares approach (Excel Solver) to either first-order, *A*(*t*) = *A*_0_*e*^−*kt*^, or second order, $A\left(t\right)=A_{0}\left({Pe}^{-k_{1}t}+\left(1-P\right)e^{-k_{2}t}\right)$, rate equations, where *A*_*0*_ equals the virus concentration at t = 0, *A(t)* equals virus concentrations at selected time points, *t* equals time and *k* terms (*k*, *k*_*1*_ and *k*_*2*_) are reaction rate constants. For second-order reactions, *P* equals the fraction of virus rapidly inactivated, *k*_*1*_ is the reaction rate constant for the rapid phase and *k*_*2*_ is the reaction rate constant for the slow phase. Half-life is defined as *T*_1/2_ = ln(2)/*k*. Comparisons of viremia levels post-challenge were made separately at each time point relative to naïve controls using ANOVA with Holm-Šídák multiple test correction, while survival comparisons were made using the Mantel-Cox log rank test (Prism v9.2.0, GraphPad Software).

## Supporting information

S1 Fig Methisazone (MZ) drives rapid, site-directed inactivation of virus while protecting neutralizing epitopes from oxidative damage observed with H_2_O_2_ alone. Comparisons were made between high concentration H_2_O_2_ (3% H_2_O_2_), a site-directed dual oxidation approach (0.0003% H_2_O_2_, 2 μM CuCl_2_, 20 μM methisazone [H_2_O_2_/CuCl_2_/MZ]) and selected subcomponents of the site-directed dual oxidation system at matched concentrations (H_2_O_2_/CuCl_2_ or MZ alone). Maintenance of neutralizing antibody binding sites was assessed using an antigen-capture ELISA comprised of a neutralizing CHIKV-specific MAb (MAb 13b8). All antigens were tested after 20 hrs of room temperature incubation and neutralizing epitope maintenance was compared to freshly thawed purified CHIKV. Inactivation half-life calculations are described in Fig 2A. The average of triplicate experiments is shown with SD error bars. The dotted line indicates the limit of quantitation.
(PDF)

S2 Fig Heat treatment of CHIKV demonstrates inactivation rates that become slower over time. Infectious CHIKV 181/25 virus titers were measured by plaque assay after heat treatment at 56°C (infected Vero cell culture supernatant to mimic research-grade material and purified virus to simulate vaccine-grade material) or 65°C (purified virus to simulate vaccine-grade material). Individual 0.25 mL aliquots of 56°C supernatant virus were transferred from room temperature to 56°C and then transferred to ice at the indicated time points to stop further heat-based inactivation. Individual 1 mL aliquots of purified virus were transferred from ice to 56°C or to 65°C for the specified periods of time before being transferred back to ice to stop heat inactivation. The dotted line indicates the limit of detection and open symbols signify time points when no detectable live virus was observed.
(PDF)

S3 Fig Cross-neutralization of heterologous CHIKV strains and other arthritogenic alphaviruses. Purified CHIKV-181/25 was inactivated with the optimized HydroVax approach (20 hours at room temperature) or formaldehyde (Form., 0.01% for 20 days at 37°C). Each vaccine antigen was formulated with alum and used to immunize mice at a high dose (2 μg, dark symbols) or low dose (0.5–0.8 μg, light symbols) on days 0 and 28, with serum samples collected at 56 days following the primary vaccination (28 days following booster vaccination). Neutralization was tested against a panel of (A) CHIKV strains representing genotypic and geographic diversity and (B) a panel of clinically relevant arthritogenic alphaviruses. Each individual data point is shown along with the geometric mean represented by a bar and the error bars represent 95% confidence intervals. Dotted lines represent the limit of detection.
(PDF)

S4 Fig GK1.5 monoclonal antibody treatment efficiently depletes murine CD4^+^ T cells. CD4^+^ T cells were measured by flow cytometry was performed on splenocytes harvested from representative mice that had been treated intraperitoneally with MAb GK1.5 (αCD4; 100 μg per dose) on days 0, 1 and 4, with splenocytes harvested at day 5.5 and stained with the rat anti-CD4 MAb, clone RM4-4, which does not compete for binding with the GK1.5 CD4-depleting antibody.
(PDF)
