# Supplementary material for: Development of a next-generation chikungunya virus vaccine based on the HydroVax platform
Source: PLoS Pathog. 2022 Jul 5;18(7):e1010695. doi: 10.1371/journal.ppat.1010695 (PMC9286250; doi:10.1371/journal.ppat.1010695)
Supplement: S1 Fig — Comparisons were made between high concentration H2O2 (3% H2O2), a site-directed dual oxidation approach (0.0003% H2O2, 2 μM CuCl2, 20 μM methisazone [H2O2/CuCl2/MZ]) and selected subcomponents of the site-directed dual oxidation system at matched concentrations (H2O2/CuCl2 or MZ alone). Maintenance of neutralizing antibody binding sites was assessed using an antigen-capture ELISA comprised of a neutralizing CHIKV-specific MAb (MAb 13b8). All antigens were tested after 20 hrs of room temperature incubation and neutralizing epitope maintenance was compared to freshly thawed purified CHIKV. Inactivation half-life calculations are described in Fig 2A. The average of triplicate experiments is shown with SD error bars. The dotted line indicates the limit of quantitation. (PDF) [file ppat.1010695.s001.pdf]

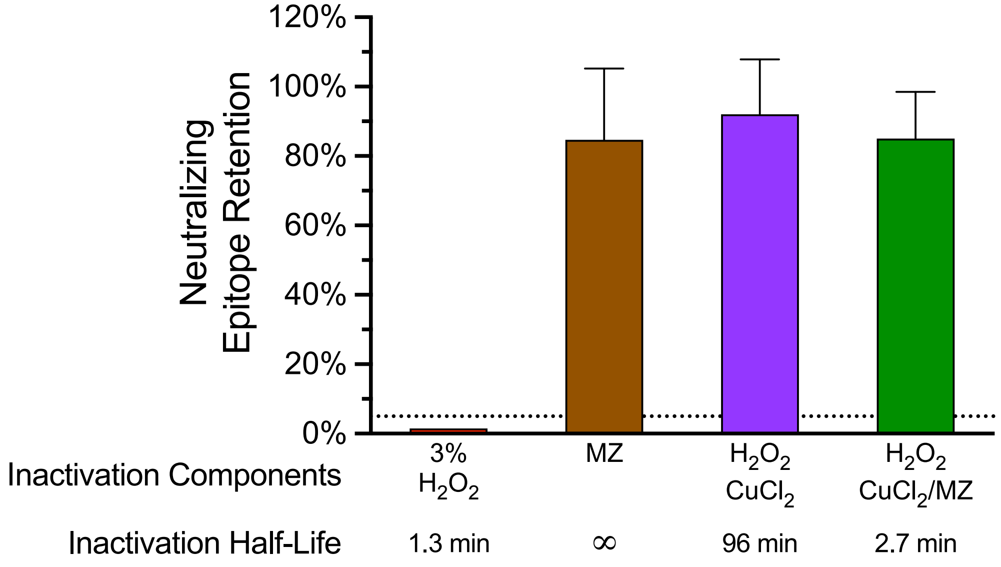

S1 Fig. Methisazone (MZ) drives rapid, site-directed inactivation of virus while protecting neutralizing epitopes from oxidative damage observed with H_2O_2 alone. Comparisons were made between high concentration H_2O_2 (3% H_2O_2), a site-directed dual oxidation approach (0.0003% H_2O_2, 2 μM $CuCl_2$, 20 μM methisazone [H_2O_2/$CuCl_2$/MZ]) and selected subcomponents of the site-directed dual oxidation system at matched concentrations (H_2O_2/$CuCl_2$ or MZ alone). Maintenance of neutralizing antibody binding sites was assessed using an antigen-capture ELISA comprised of a neutralizing CHIKV-specific MAb (MAb 13b8). All antigens were tested after 20 hrs of room temperature incubation and neutralizing epitope maintenance was compared to freshly thawed purified CHIKV. Inactivation half-life calculations are described in Fig 2A. The average of triplicate experiments is shown with SD error bars. The dotted line indicates the limit of quantitation.
