# Supplementary material for: Development of a next-generation chikungunya virus vaccine based on the HydroVax platform
Source: PLoS Pathog. 2022 Jul 5;18(7):e1010695. doi: 10.1371/journal.ppat.1010695 (PMC9286250; doi:10.1371/journal.ppat.1010695)
Supplement: S2 Fig — Infectious CHIKV 181/25 virus titers were measured by plaque assay after heat treatment at 56°C (infected Vero cell culture supernatant to mimic research-grade material and purified virus to simulate vaccine-grade material) or 65°C (purified virus to simulate vaccine-grade material). Individual 0.25 mL aliquots of 56°C supernatant virus were transferred from room temperature to 56°C and then transferred to ice at the indicated time points to stop further heat-based inactivation. Individual 1 mL aliquots of purified virus were transferred from ice to 56°C or to 65°C for the specified periods of time before being transferred back to ice to stop heat inactivation. The dotted line indicates the limit of detection and open symbols signify time points when no detectable live virus was observed. (PDF) [file ppat.1010695.s002.pdf]

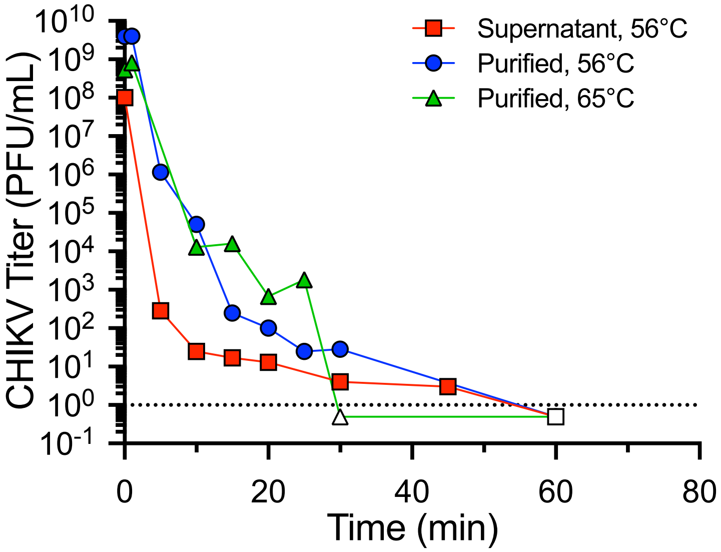

S2 Fig. Heat treatment of CHIKV demonstrates inactivation rates that become slower over time. Infectious CHIKV 181/25 virus titers were measured by plaque assay after heat treatment at 56°C (infected Vero cell culture supernatant to mimic research-grade material and purified virus to simulate vaccine-grade material) or 65°C (purified virus to simulate vaccine-grade material). Individual 0.25 mL aliquots of 56°C supernatant virus were transferred from room temperature to 56°C and then transferred to ice at the indicated time points to stop further heat-based inactivation. Individual 1 mL aliquots of purified virus were transferred from ice to 56°C or to 65°C for the specified periods of time before being transferred back to ice to stop heat inactivation. The dotted line indicates the limit of detection and open symbols signify time points when no detectable live virus was observed.
