# Supplementary material for: Development of a next-generation chikungunya virus vaccine based on the HydroVax platform
Source: PLoS Pathog. 2022 Jul 5;18(7):e1010695. doi: 10.1371/journal.ppat.1010695 (PMC9286250; doi:10.1371/journal.ppat.1010695)
Supplement: S3 Fig — Purified CHIKV-181/25 was inactivated with the optimized HydroVax approach (20 hours at room temperature) or formaldehyde (Form., 0.01% for 20 days at 37°C). Each vaccine antigen was formulated with alum and used to immunize mice at a high dose (2 μg, dark symbols) or low dose (0.5–0.8 μg, light symbols) on days 0 and 28, with serum samples collected at 56 days following the primary vaccination (28 days following booster vaccination). Neutralization was tested against a panel of (A) CHIKV strains representing genotypic and geographic diversity and (B) a panel of clinically relevant arthritogenic alphaviruses. Each individual data point is shown along with the geometric mean represented by a bar and the error bars represent 95% confidence intervals. Dotted lines represent the limit of detection. (PDF) [file ppat.1010695.s003.pdf]

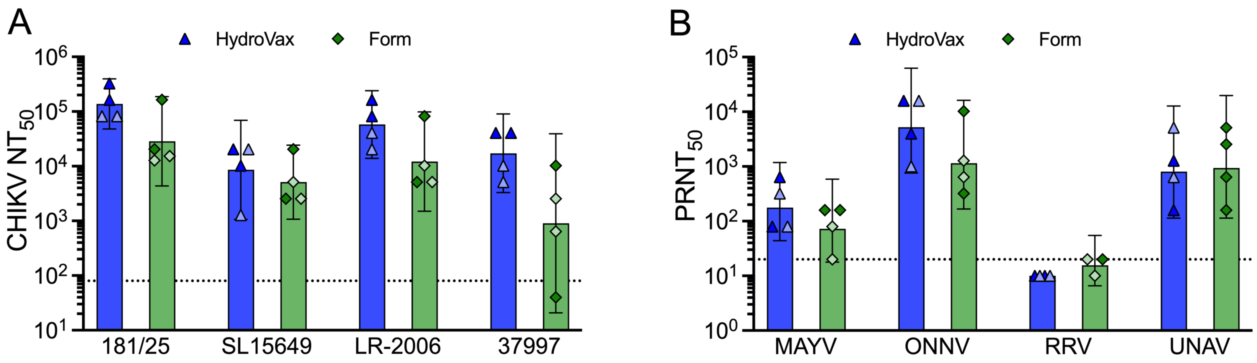

S3 Fig. Cross-neutralization of heterologous CHIKV strains and other arthritogenic alphaviruses. Purified CHIKV-181/25 was inactivated with the optimized HydroVax approach (20 hours at room temperature) or formaldehyde (Form., 0.01% for 20 days at 37°C). Each vaccine antigen was formulated with alum and used to immunize mice at a high dose (2 µg , dark symbols) or low dose (0.5 – 0.8 µg, light symbols) on days 0 and 28, with serum samples collected at 56 days following the primary vaccination (28 days following booster vaccination). Neutralization was tested against a panel of (A) CHIKV strains representing genotypic and geographic diversity and (B) a panel of clinically relevant arthritogenic alphaviruses. Each individual data point is shown along with the geometric mean represented by a bar and the error bars represent 95% confidence intervals. Dotted lines represent the limit of detection.
