# Supplementary material for: Development of a next-generation chikungunya virus vaccine based on the HydroVax platform
Source: PLoS Pathog. 2022 Jul 5;18(7):e1010695. doi: 10.1371/journal.ppat.1010695 (PMC9286250; doi:10.1371/journal.ppat.1010695)
Supplement: S4 Fig — CD4+ T cells were measured by flow cytometry was performed on splenocytes harvested from representative mice that had been treated intraperitoneally with MAb GK1.5 (αCD4; 100 μg per dose) on days 0, 1 and 4, with splenocytes harvested at day 5.5 and stained with the rat anti-CD4 MAb, clone RM4-4, which does not compete for binding with the GK1.5 CD4-depleting antibody. (PDF) [file ppat.1010695.s004.pdf]

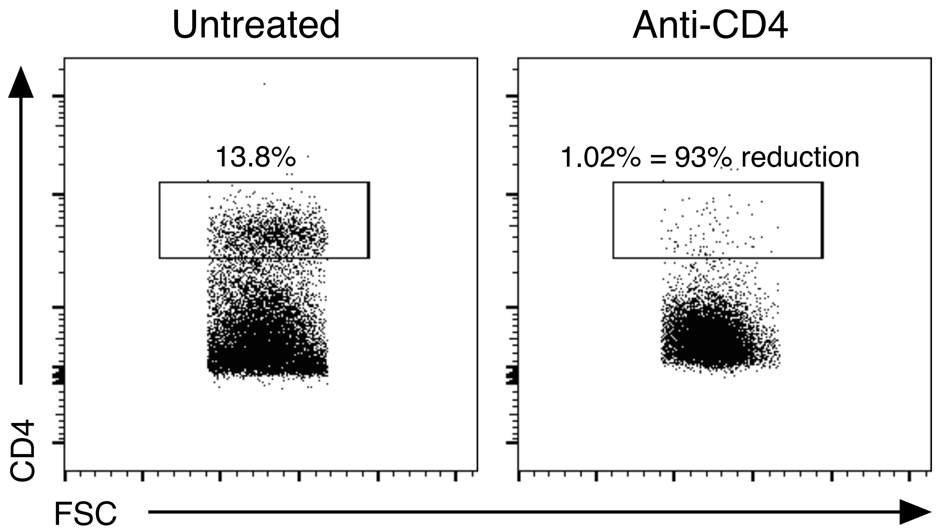

S4 Fig. GK1.5 monoclonal antibody treatment efficiently depletes murine CD4$^+$ T cells. CD4$^+$ T cells were measured by flow cytometry was performed on splenocytes harvested from representative mice that had been treated intraperitoneally with MAb GK1.5 (αCD4; 100 μg per dose) on days 0, 1 and 4, with splenocytes harvested at day 5.5 and stained with the rat anti-CD4 MAb, clone RM4-4, which does not compete for binding with the GK1.5 CD4-depleting antibody.
